# Development and Validation of a Questionnaire to Measure Adherence to a Mediterranean-Type Diet in Youth

**DOI:** 10.3390/nu16162754

**Published:** 2024-08-18

**Authors:** Yu-Jin Kwon, Young-Hwan Park, Yae-Ji Lee, Li-Rang Lim, Ji-Won Lee

**Affiliations:** 1 Department of Family Medicine, Yongin Severance Hospital, College of Medicine, Yonsei University, Yongin 16995, Republic of Korea; digda3@yuhs.ac; 2 Incheon Grand Internal Medicine Clinic, Incheon 22184, Republic of Korea; withimok@naver.com; 3 Department of Biostatistics and Computing, Yonsei University, Seoul 03722, Republic of Korea; ysbiostat@yuhs.ac; 4 Department of Family Medicine, Severance Hospital, College of Medicine, Yonsei University, Seoul 03722, Republic of Korea; erang0422@yuhs.ac; 5 Institute for Innovation in Digital Healthcare, Yonsei University, Seoul 03722, Republic of Korea

**Keywords:** Mediterranean diet, adherence, questionnaire, youth, validation

## Abstract

Proper nutrition during childhood is crucial for preventing chronic diseases and ensuring optimal growth. This study aimed to develop and validate the Korean version of the KIDMED (K-KIDMED) questionnaire to accurately measure Mediterranean diet (MD) adherence among Korean children and adolescents. A total of 226 parents, representing their children and adolescents, completed the K-KIDMED, a 112-item food frequency questionnaire (FFQ), and a 24-h dietary recall method through an anonymous online survey. The K-KIDMED comprised 11 questions, with five excluded from the original scoring as they did not apply to the FFQ. Scores were categorized into three levels of adherence to the MD: low (1 or less), average (2–4), and good (5 or more). The agreement between total MD scores from the Korean version of the Mediterranean diet adherence screener and the FFQ was moderate (intraclass correlation coefficient = 0.455, 95% confidence interval: 0.346, 0.553). Among the 226 children and adolescents, 36.7% had low adherence to the KIDMED, 43.3% had intermediate adherence, and 19.9% had good adherence. Higher K-KIDMED scores were correlated with greater intakes of fiber, vitamin K, vitamin B6, and potassium (all *p* < 0.05). We developed the K-KIDMED as a valid tool to assess MD adherence in Korean children and adolescents.

## 1. Introduction

Proper childhood nutrition is essential for preventing chronic diseases and ensuring optimal growth. Establishing healthy dietary habits early in life leads to lifelong benefits [1], while poor nutrition can adversely impact physical and mental development [2]. Therefore, it is crucial to evaluate the nutritional status of children and adolescents, provide appropriate nutritional information, and offer education to transform their current dietary behaviors into healthy habits for adulthood [3].

The Mediterranean diet (MD) is recognized as one of the healthiest dietary patterns globally. It emphasizes high consumption of fruits, vegetables, legumes, olive oil, nuts, and cereals; moderate-to-high intake of fish and dairy products; and low intake of saturated fats, sweets, and red and processed meats [4,5]. Adherence to the MD significantly reduces overall mortality and offers numerous health benefits, including the prevention of obesity, type 2 diabetes, cardiovascular diseases, and cancers in adults [6,7]. Additionally, the MD contributes to better cognitive and mental health outcomes [8,9].

While most research on the MD focuses on adults, emerging evidence suggests that the MD may also promote health in children. The MD is known to reduce childhood obesity, prevent metabolic syndrome, and be effective in managing attention-deficit/hyperactivity disorder and depression in children and adolescents [9,10,11,12].

Recent research emphasizes the widespread use of the KIDMED questionnaire, developed in 2004 by Serra-Majem et al., to assess adherence to the MD among children and adolescents, particularly in the Mediterranean region and Europe [13]. The KIDMED index, comprising 16 questions, is a frequently employed tool for evaluating dietary adherence in young populations [13]. High adherence to the MD is also associated with better physical activity, sleep quality, body image satisfaction, and overall health-related quality of life in children and adolescents [14,15].

Due to the diverse eating habits across different countries and ethnicities, applying the existing KIDMED index to non-Mediterranean populations without proper validation is challenging. Consequently, there are limited studies using validated questionnaires to estimate adherence to the MD in Asian children and adolescents [16,17]. Specifically, there is a lack of standardized tools for assessing MD adherence among Korean children and adolescents, as the current KIDMED survey is not well-suited to Korean food culture.

Therefore, the objective of this study was to develop a Korean MD adherence assessment tool for children and adolescents (K-KIDMED) and validate its accuracy in assessing adherence to the MD among Korean youth.

## 2. Materials and Methods

### 2.1. Study Population

This cross-sectional study was conducted with Korean parents of children and adolescents. Participants voluntarily agreed to participate, and the study included a single child from each household. The survey was conducted using survey panels from dataSpring (https://ko.d8aspring.com/contact between June and October 2023. A total of 226 parents completed the K-KIDMED, a 112-item food frequency questionnaire (FFQ), and a 24-h recall method through an anonymous online survey, reporting on behalf of their children’s dietary habits. All participants provided informed consent, and the study was approved by the Institutional Review Board of Severance Hospital (IRB No 4-2023-0351, approval date: 22 May 2023). This study adhered to the principles of the Declaration of Helsinki.

### 2.2. Data Collection, Anthropometry, and Nutritional Assessment

Data were collected during the online survey using an anonymous, self-administered questionnaire. Weight and height were self-reported. Body mass index (BMI) (kg/m^2^) was calculated to determine BMI-for-age percentiles using the Korean Center for Disease Control and Prevention (CDC) growth charts [18]: underweight (<5th percentile), normal weight (5th–85th percentile), overweight (85th–95th percentile), and obese (≥95th percentile).

The main survey questionnaire included: (1) K-KIDMED (Table S1), (2) FFQ (Table S2) [19], and (3) the 24-h recall method.

The 112-item FFQ was previously developed by the Korean National Health and Nutritional Survey team of the Korean CDC. The FFQ presented a list of foods frequently consumed by Koreans and investigated the frequency and quantity of consumption over the past year. This FFQ was developed for adults and previously validated for accuracy and reliability [19]. Therefore, the FFQ was administered, excluding alcohol-related items, to understand the overall dietary habits of children and adolescents. Food intake frequency was categorized into nine groups: never or rarely, once a month, twice or thrice a month, 1–2 times a week, 3–4 times a week, 5–6 times a week, once a day, twice a day, or ≥3 times a day. Foods were grouped to obtain a corresponding score to that of the K-KIDMED. A detailed explanation of the grouping method is presented in the Supplementary Materials.

All participants were required to complete the 24-h dietary recall survey through a web-based application. Nutrient intakes were calculated using CAN-Pro 5.0, developed by the Korean Nutrition Society (http://www.kns.or.kr, accessed on 18 July 2024).

The proportion of carbohydrates and protein was calculated as carbohydrate intake (g) × 4 kcal/total energy intake (kcal/day) and protein intake (g) × 4 kcal/total energy intake (kcal/day), respectively. The proportion of fats (fat, saturated fat, monounsaturated fat, polyunsaturated fat, omega-3, and omega-6) was calculated as the intake of each fat type (g) × 9 kcal/total energy intake (kcal/day). The measurement units of vitamins were recorded based on the 9th revision of the Korean food composition table [20].

### 2.3. Development of K-KIDMED

Serra-Majem et al. developed a 16-item measure of adherence to the MD, known as KIDMED [13]. Permission to use the 16-item KIDMED was obtained from the authors via email. The KIDMED was translated into Korean and reviewed by two physicians and four nutritionists. Before the commencement of the main study, a pilot study was conducted to assess the comprehensibility and the time required to complete the K-KIDMED questionnaire. During this pilot phase, two questions were found to be challenging for application in the Korean population: “Consumes pasta or rice almost every day [five or more times per week]?” and “Has cereals or grains [bread, etc.] for breakfast?” As a result, an expert group, including two physicians and four nutritionists, modified these questions for better relevance and clarity. The modified question was: “I eat multigrain rice, rye bread, barley bread, etc., more than five times a week”. This modification was intended to better align with Korea’s dietary habits and food availability. All questions were independently reviewed by two Korean translators and then translated back into English by two native speakers who were not involved in the study. An expert committee, including physicians, nutritionists, and a methodologist, collaborated to create a piloted version of the translated questionnaire. Cognitive interviews were conducted with a small sample of 15 parents of children and adolescents to ensure that respondents’ interpretations aligned with the intended meanings. This process was repeated three times before finalizing the translated version of the questionnaire. Nineteen experts evaluated the content validity, and the final adapted questionnaire was reviewed and approved by the study team.

Initially, we created 16 questions to adapt the KIDMED score. However, five questions were excluded from scoring as they did not apply to the FFQ questionnaire. The following questions were excluded: ‘I eat multigrain rice, rye bread, and barley bread for breakfast’, ‘I usually eat food with olive oil and perilla oil’, ‘I do not have breakfast more than 2–3 times a week’, ‘I eat dairy products for breakfast (e.g., white milk, scooped yogurt, etc.)’, and ‘I eat sweet bread, cakes, doughnuts, and pastries (croissants, pies, etc.) for breakfast’. Since olive oil is not used in Korean cuisine, we excluded questions about olive oil from the assessment.

A score of 1 was assigned in the following cases: for Q1, “I consume a fruit or fruit juice every day”; for Q2, “I consume a fruit or fruit juice more than twice a day”; for Q3, “I eat fresh or cooked vegetables more than once a day (e.g., kimchi, cucumber, spinach salad, bean sprout salad, salad, etc.)”; for Q4, “I eat fresh or cooked vegetables more than twice a day”; for Q5, “I consume fish or seafood more than 2–3 times a week (e.g., mackerel, saury, salmon, flounder, flatfish, squid, shrimp, webfoot octopus, etc.)”; for Q7, “I eat foods containing beans more than once a week (e.g., tofu, soft tofu, bean sauce, soymilk, etc.)”; for Q8, “I eat multigrain rice, rye bread, barley bread, etc., more than five times a week”; for Q9, “I eat nuts more than 2–3 times a week (e.g., peanuts, walnuts, almonds, pistachios, macadamia, etc.)”; and for Q10, “I eat more than two yogurts or two slices of cheese a day.”

A score of −1 was assigned in the following cases: for Q6, “I eat fast food or instant food more than once a week (e.g., hamburgers, pizza, hot dogs, chicken, ramen, convenience store lunch boxes, etc.)” and for Q11, “I eat sweet snacks more than twice a day (e.g., beverages, snacks, candy, jelly, chocolate, etc.)”.

If the criterion was not met, 0 points were allotted; therefore, the sum of the K-KIDMED score ranged from −2 to 8 points. The KIDMED score was divided into three categories according to tertiles: (1) low adherence to the MD: K-KIDMED score of 1 or less; (2) average adherence to the MD: K-KIDMED score between 2 and 4; and (3) good adherence to the MD: K-KIDMED score of 5 or more.

### 2.4. Sample Size

Based on various references [21], we initially aimed for a sample size of 240 participants (15:1 ratio), with a minimum of 160 participants (10:1 ratio). However, due to the nature of the online survey, we were able to effectively utilize data from 226 participants, demonstrating our research’s adaptability and flexibility.

### 2.5. Statistical Analysis

We tested the normality of the data distribution using both graphical and statistical methods. First, we visually inspected histograms and Q-Q (quantile-quantile) plots of the variables to assess the overall shape and distribution. In addition, we performed the Kolmogorov–Smirnov test, a widely used statistical test for normality. This test was applied to each variable to determine if the data deviated significantly from a normal distribution. Continuous variables are presented as mean ± standard deviation (SD) or median (interquartile range), while categorical variables are expressed as numbers (%). We identified the FFQ items corresponding to the K-KIDMED. Binary scores were assigned to each K-KIDMED question, and absolute agreement was assessed using Cohen’s kappa coefficient (k) [22]. Cohen’s kappa coefficient (k) values were interpreted as follows: 0–0.2, poor agreement; 0.2–0.4, fair agreement; 0.4–0.6, moderate agreement; 0.6–0.8, good agreement; and 0.8–1, very good agreement. The intraclass correlation coefficient (ICC) was employed to determine the absolute agreement between the total MD adherence scores measured using the K-KIDMED and FFQ [23]. Based on the guidelines suggested by Cicchetti, an ICC of 0.455 can be interpreted as fair agreement. Intraclass correlation (ICC) values were interpreted as follows: less than 0.40, poor agreement; 0.40–0.59, fair agreement; 0.60–0.74, good agreement; and 0.75–1.00, excellent agreement [24].

Agreement between the total scores from the K-KIDMED questionnaire and equivalent FFQ questions was examined using Bland–Altman analysis, which assesses bias between mean differences and estimates an agreement interval that includes 95% of the differences. The K-KIDMED score was divided into tertiles (T): T1 (−2 to 1), T2 (2–4), and T3 (5–8). Clinical characteristics and nutritional status of the study population, stratified by K-KIDMED score tertiles, were compared using one-way analysis of variance for normally distributed variables and the Kruskal–Wallis test for non-normally distributed variables. Proportions were compared using chi-square tests. Regarding nutritional characteristics according to the K-KIDMED questions, binary scores were compared using Student’s t-test or the Mann–Whitney U test. All *p*-values were based on two-sided tests, and values below 0.05 were considered statistically significant. All analyses were conducted using R, version 4.3.0 (R Foundation for Statistical Computing, Vienna, Austria, http://www.R-project.org (accessed on 18 July 2024)).

## 3. Results

Overall, the average K-KIDMED score was 2.49 ± 2.19, while the FFQ had an average score of 3.48 ± 1.96. When analyzed by gender, males scored an average of 2.75 ± 2.29 on the K-KIDMED and 3.71 ± 1.91 on the FFQ. In comparison, females scored an average of 2.13 ± 2.00 on the K-KIDMED and 3.16 ± 1.99 on the FFQ. Table 1 presents the clinical characteristics of the study population, consisting of 132 boys and 94 girls, with a mean age of 9.1 ± 2.3 years and a mean BMI of 17.5 ± 3.4 kg/m^2^. No significant differences in age, sex, or BMI were observed across the K-KIDMED tertiles.

**Figure 1:**
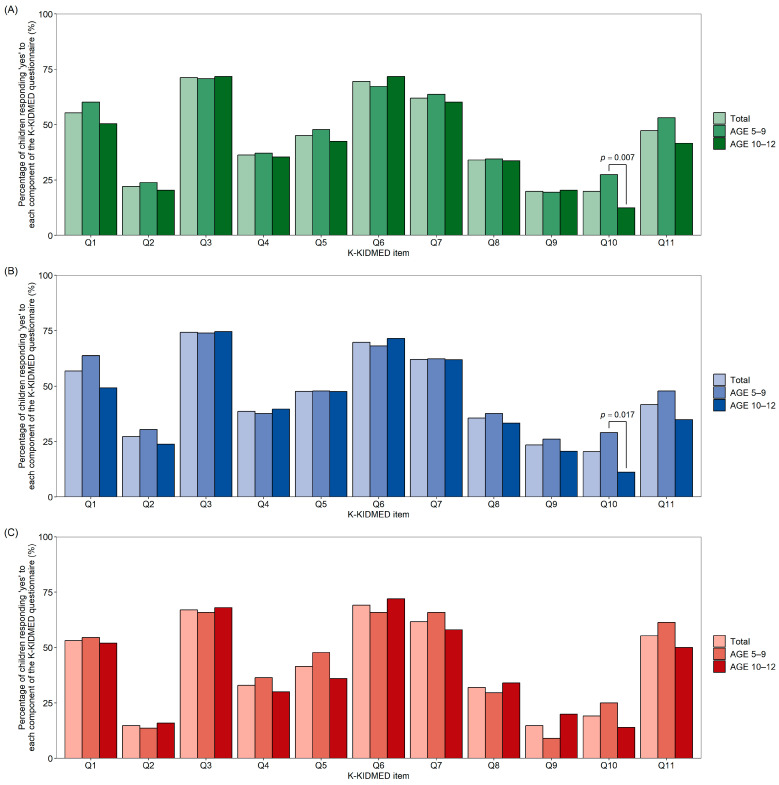
The proportion of “yes” responses for each item across different age groups. (**A**) total, (**B**) male, and (**C**) female.

Figure 1A–C illustrates the proportion of “yes” responses for each item in the total sample and across different age groups (5–9 years and 10–12 years) by sex, respectively. Q10 (“I eat more than two yogurts or two slices of cheese a day”) showed a higher percentage of “yes” responses in the 5–9-year-old group than in the 10–12-year-old group, particularly among total and males. Figure S1 shows the proportion of “yes” responses for each KIDMED item across different age groups. Figures S2 and S3 show the proportion of “yes” responses for each item across different BMI categories.

**Figure 2:**
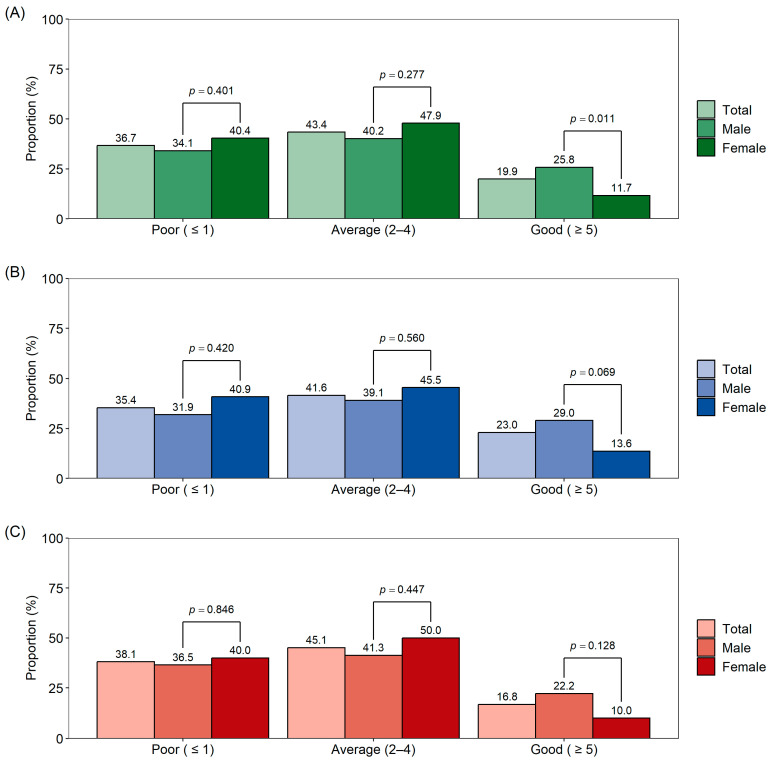
K-KIDMED test and K-KIDMED index values by age group and sex (**A**) total, (**B**) 5–9 years, and (**C**) 10–12 years.

Figure 2 shows the K-KIDMED test and K-KIDMED index values by age group and sex. Among the total study population (*n* = 226), 36.9% had low adherence to the KIDMED (“poor”: ≤1), 43.4% had intermediate adherence (“average”: 2–4), and 19.9% had good adherence (“good”: ≥5). There were no significant differences in K-KIDMED scores between sexes, except in the good adherence group (Figure 2A). When divided into age groups (5–9 years, Figure 2B; 10–12 years, Figure 2C), the K-KIDMED score showed a tendency to decrease with age, although this trend did not reach statistical significance (*p* = 0.08).

Table 2 presents the nutritional characteristics of the study population according to K-KIDMED score tertiles. The intake of fiber, vitamin K, vitamin B6, and potassium was significantly higher in the group with a higher K-KIDMED score (*p* < 0.05). Analysis of nutritional characteristics based on responses to the K-KIDMED questionnaire revealed significant differences (Table S3). Specifically, for items Q2, Q3, and Q9, those who responded “yes” had significantly higher fiber intake. Similarly, individuals who responded “yes” to Q8 had significantly higher vitamin K, potassium, and magnesium intake. Additionally, those who responded “yes” to Q2 had significantly higher vitamin B6 intake.

When analyzed using FFQ score tertiles, the intake of fiber, vitamin D, vitamin C, thiamine, folic acid, vitamin B12, calcium, potassium, magnesium, zinc, copper, and omega-3 was significantly higher in the good adherence group (all *p* < 0.05). Although the statistical significance of vitamin K decreased, it remained higher in the good adherence group (*p* = 0.066) (Table S4).

Table 3 presents absolute agreement values between the K-KIDMED and FFQ. Question 8 demonstrated a moderate strength of agreement (kappa = 0.405); questions 1, 3, 4, and 7 exhibited a fair strength of agreement (kappa = 0.310, 0.358, 0.362, and 0.352, respectively); and questions 2, 5, 6, and 9 displayed weak strength of agreement (kappa = 0.144, 0.148, 0.081, and 0.186, respectively). The concordance between the total K-KIDMED and FFQ scores was fair (ICC = 0.455).

**Figure 3:**
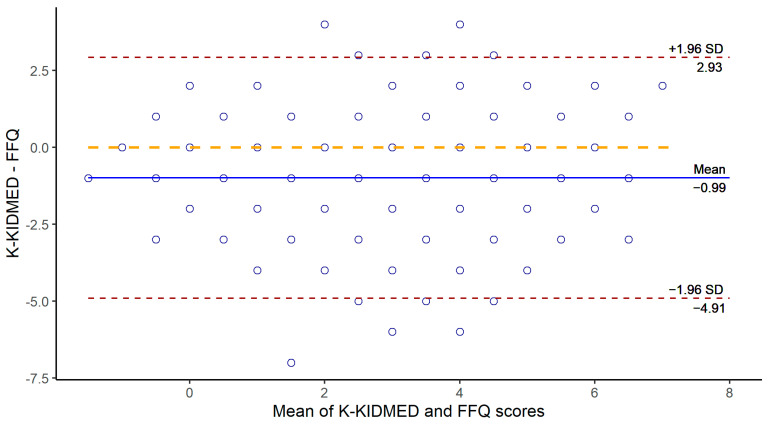
Bland–Altman plot for agreement between the total K-KIDMED and FFQ scores. FFQ, food frequency questionnaire. The Bland-Altman plot is a graphical method for evaluating the agreement between two measurement methods by plotting the differences between their measurements against their mean.

The agreement between total K-KIDMED and FFQ scores was analyzed using a Bland–Altman plot (Figure 3).

## 4. Discussion

Healthy lifestyle habits formed during childhood and adolescence significantly influence lifelong health by shaping dietary patterns and reducing susceptibility to chronic diseases [25]. Emerging evidence suggests that the MD is beneficial for children’s health, leading to its widespread use in public health programs aimed at promoting healthy eating habits among children, both in Mediterranean and non-Mediterranean countries [26]. The level of adherence to the MD can be assessed using the KIDMED index, which has shown a positive correlation with improved nutrient intake [14].

In this study, we validated an 11-item K-KIDMED, adapted to reflect Korean dietary habits and portion sizes suitable for Korean children. Among the 11 questions, six showed moderate or fair kappa values when comparing the total K-KIDMED with FFQ scores. The absolute agreement level between the total MD scores from the Korean version of the Mediterranean Diet Adherence Screener and the FFQ was moderate, with a value of 0.455 (95% confidence interval: 0.346, 0.553), comparable to previous studies validating the KIDMED in Portugal and Colombia [27,28]. To revise KIDMED, we used a semi-quantitative FFQ from the Korean CDC, excluding alcohol items, to assess dietary habits in children and adolescents.

In our study, K-KIDMED scores did not show statistically significant differences based on BMI categories, consistent with the findings of several other studies [29,30,31]. Additionally, our results demonstrated a relatively low prevalence of good adherence to the MD across the entire sample. Generally, 40–50% of children and adolescents exhibit average to high adherence to the MD in the Mediterranean region [31]. However, similar low adherence rates to those found in our study have been reported in various countries [13,32,33,34], underscoring the influence of geographical region. For example, a recent U.S. study reported that 81.4% of adolescents had poor adherence, 17.8% had average adherence, and only 0.75% had good adherence [35]. In recent decades, dietary habits in Asian countries, including Korea, have shifted from traditional low-fat, high-fiber diets to fast foods and sugary beverages, with reduced vegetable intake [36,37]. This dietary shift has significantly impacted nutritional status and public health [38,39], contributing to nutritional imbalances among Korean children and adolescents, which is a critical concern during their growth period [40]. For instance, obesity rates in Korean children aged 6–18 years increased from 8.7% in 2007 to 15.0% in 2017, highlighting the need for targeted interventions [41]. Therefore, developing strategies to enhance dietary patterns and reduce the disease burden is essential. Promoting adherence to the MD could be a beneficial step in these efforts.

Interestingly, our analysis showed that individuals with higher K-KIDMED scores had significantly greater intakes of fiber, vitamin K, vitamin B6, and potassium. These findings support the index’s validity, as higher scores—indicating better adherence to the MD—were linked to improved nutritional adequacy, particularly in vitamins and minerals [42]. Childhood dietary fiber intake is associated with numerous health benefits, including promoting regular bowel movements and reducing the risk of adult-onset diabetes, certain cancers, and cardiovascular disease in the future [43]. However, according to the 2020 dietary reference intake for Koreans, fiber consumption was below the recommended levels of 25–30 g/day for boys and 20–25 g/day for girls, highlighting the need for nutritional interventions to promote fiber-rich diets [44].

Additionally, children require more vitamin K intake than adults due to common health issues and treatments in pediatrics, with deficiencies linked to bruising, bleeding, and poor bone health [45]. Supplementation with vitamin K, especially vitamin K2, supports coagulation, bone mineralization, and cardiovascular health, helping to prevent arterial calcification from a young age [46]. Dietary sources rich in potassium and vitamin B6 include whole and unrefined grains, seeds, nuts, and green leafy vegetables, which are the main components of the MD [47]. Potassium intake is associated with improved nutritional quality and a reduced risk of metabolic syndrome [48]. Vitamin B6 plays a crucial role in amino acid metabolism, neurotransmitter synthesis, and maintaining normal cognitive and immune functions [49]. Our data primarily derive from cross-sectional studies; hence, further research is essential to explore the effectiveness of K-KIDMED in assessing adherence to the MD, particularly in relation to intakes of fiber, vitamin K, vitamin B6, and potassium, and their associations with biological outcomes.

Our study has several limitations. First, the small sample size limits generalizability, and larger representative studies are needed for validation. Second, nutritional information relied on parent-reported data, which carries the risk of misreporting and bias, potentially leading to overestimation or underestimation. Third, we did not collect data on other factors such as socioeconomic status, exercise, and sleep habits, limiting the scope of our findings. Fourth, the FFQ is not considered a gold-standard measure of dietary intake. While the FFQ provides valuable insights, weighed food diaries, which capture detailed meal-by-meal intake, are more accurate for validating dietary intake. A weighted dietary diary would have addressed missing questions and provided a more robust comparison. Future studies should employ more rigorous dietary intake methods, such as weighted food diaries, to validate the K-KIDMED tool more effectively. Fifth, the current K-KIDMED score does not account for olive oil, a key component of the Mediterranean diet. However, in Korea, perilla oil and sesame oil are commonly used in vegetable dishes [50]. Sixth, omitting five items from the original KIDMED test in the K-KIDMED could be a potential source of bias and explain the low scores observed. Finally, we were unable to conduct a test-retest for validation. Future studies should address these limitations to further validate the accuracy of the data collected.

Despite these limitations, our study has notable strengths. To our knowledge, it is the first to develop a questionnaire assessing adherence to the MD among Korean children. Additionally, the K-KIDMED index has been validated against other dietary assessment tools, such as the FFQ, demonstrating reliable agreement between the K-KIDMED and FFQ.

## 5. Conclusions

We developed the K-KIDMED as a valid tool to assess Korean children’s adherence to the MD. This tool can help monitor dietary patterns and provide guidelines for healthy eating. Our findings indicate low adherence to MD principles, highlighting the need for improved dietary patterns. Extensive population-based studies are needed to further validate the usefulness of K-KIDMED.

## Figures and Tables

**Table 1:** Baseline characteristics according to K-KIDMED tertiles (*N* = 226).

			K-KIDMED		
	Overall	T1	T2	T3	*p*-Value
*n*	226	83	98	45	
Sex, *n* (%)					0.032
Male	132 (58.4%)	45 (54.2%)	53 (54.1%)	34 (75.6%)	
Female	94 (41.6%)	38 (45.8%)	45 (45.9%)	11 (24.4%)	
Age, years	9.1 ± 2.2	9.4 ± 2.1	9.0 ± 2.2	8.9 ± 2.4	0.439
Age groups, *n* (%)					
5	21 (9.3%)	4 (4.8%)	13 (13.3%)	4 (8.9%)	
6	15 (6.6%)	4 (4.8%)	7 (7.1%)	4 (8.9%)	
7	18 (8.0%)	9 (10.8%)	4 (4.1%)	5 (11.1%)	
8	34 (15.0%)	14 (16.9%)	10 (10.2%)	10 (22.2%)	
9	25 (11.1%)	9 (10.8%)	13 (13.3%)	3 (6.7%)	
10	38 (16.8%)	14 (16.9%)	21 (21.4%)	3 (6.7%)	
11	38 (16.8%)	11 (13.3%)	21 (21.4%)	6 (13.3%)	
12	37 (16.4%)	18 (21.7%)	9 (9.2%)	10 (22.2%)	
Height, cm	135.9 ± 16.2	136.9 ± 16.0	135.2 ± 16.8	135.5 ± 15.6	0.764
Body weight, kg	34.8 ± 11.6	34.9 ± 11.2	34.6 ± 11.2	35.1 ± 13.2	0.972
BMI, kg/m^2^	18.4 ± 3.5	18.2 ± 3.2	18.6 ± 3.7	18.6 ± 3.7	0.751
BMI classification, *n* (%)					0.518
Underweight	17 (7.5%)	9 (10.8%)	6 (6.1%)	2 (4.4%)	
Normal weight	156 (69.0%)	55 (66.3%)	68 (69.4%)	33 (73.3%)	
Overweight	24 (10.6%)	6 (7.2%)	14 (14.3%)	4 (8.9%)	
Obese	29 (12.8%)	13 (15.7%)	10 (10.2%)	6 (13.3%)	

BMI, body mass index. T1: K-KIDMED score Tertile 1 (range: −2 to 1), T2: K-KIDMED score Tertile 2 (range: 2 to 4), and T3: K-KIDMED score Tertile 3 (range: 5 to 8). Data are expressed as mean ± standard deviation (SD) or *n* (%).

**Table 2:** Nutritional characteristics of the study population according to K-KIDMED score tertiles.

		K-KIDMED		
Characteristic	T1	T2	T3	*p*-Value
*n*	83	98	45	
Total energy, kcal	1688.5 ± 546.7	1720.8 ± 486.2	1685.7 ± 495.3	0.889
Carbohydrate, g	232.1 ± 74.8	234.1 ± 67.4	237.6 ± 61.9	0.914
Fat, g	54.6 (32.4–66.3)	49.6 (40.2–67.3)	49.6 (33.3–68.7)	0.746
Protein, g	61.6 (46.7–81.2)	64.2 (48.2–80.1)	58.8 (51.5–71.1)	0.451
Fiber, g	14.0 (11.0–18.2)	15.7 (12.7–20.0)	17.2 (13.2–22.0)	0.015
Vitamin A, μg RAE	275.1 (209.5–442.2)	295.7 (178.5–392.8)	256.9 (161.0–363.0)	0.472
Retinol, μg	119.7 (59.4–229.6)	88.4 (49.5–169.2)	81.2 (31.2–156.6)	0.090
β-Carotene, μg	1640.8 (674.8–2997.3)	1488.3 (881.1–3005.8)	1895.9 (975.7–3288.4)	0.559
Vitamin D, μg	3.0 (1.3–4.5)	2.1 (1.1–5.3)	2.5 (0.9–5.1)	0.660
Vitamin E, mg	12.3 (7.3–18.0)	12.0 (9.2–15.2)	12.7 (7.1–17.9)	0.911
Vitamin K, μg	53.1 (23.3–99.5)	63.4 (34.2–152.0)	88.1 (50.0–166.8)	0.033
Vitamin C, mg	28.8 (13.3–53.2)	38.3 (24.2–62.9)	42.0 (22.4–70.6)	0.080
Thiamine, mg	1.3 (1.0–1.9)	1.4 (1.1–2.0)	1.3 (1.1–1.9)	0.260
Riboflavin, mg	1.4 ± 0.6	1.3 ± 0.5	1.3 ± 0.5	0.664
Niacin, mg	10.4 (7.0–14.5)	11.1 (8.6–13.7)	9.9 (7.3–11.3)	0.109
Vitamin B6, mg	1.1 (0.8–1.4)	1.3 (1.0–1.6)	1.2 (1.0–1.6)	0.043
Folic acid, μg	284.3 (188.0–426.8)	329.1 (246.9–422.9)	373.9 (241.3–443.5)	0.246
Vitamin B12, μg	4.2 (2.6–6.8)	4.1 (2.6–8.2)	4.1 (3.1–7.2)	0.936
Calcium, mg	415.6 (264.1–550.1)	390.1 (253.3–561.2)	380.8 (256.6–496.6)	0.739
Phosphate, mg	932.5 ± 374.0	978.5 ± 335.1	906.8 ± 318.5	0.462
Sodium, mg	2976.8 ± 1295.4	3225.2 ± 1294.8	2976.3 ± 1304.2	0.367
Potassium, mg	1905.2 (1292.4–2419.0)	2158.3 (1689.9–2719.0)	2033.4 (1714.4–2601.6)	0.040
Magnesium, mg	91.3 (55.1–115.0)	95.6 (59.0–138.1)	104.7 (74.2–139.7)	0.080
Iron, mg	11.0 (7.7–14.1)	11.7 (7.8–13.9)	12.3 (8.9–14.9)	0.457
Zinc, mg	7.4 (5.3–9.8)	8.8 (6.7–11.2)	8.4 (6.5–10.1)	0.062
Copper, ug	355.1 (258.7–527.6)	420.4 (306.1–511.5)	431.3 (270.8–565.9)	0.377
Total cholesterol, mg	279.1 (158.2–398.5)	241.4 (114.3–351.2)	227.2 (88.9–390.9)	0.223
Saturated fatty acid, g	9.3 (5.7–14.6)	9.2 (5.6–13.6)	9.7 (7.1–15.1)	0.850
MUFA, g	8.3 (5.2–14.5)	9.3 (6.1–13.1)	9.6 (5.2–14.3)	0.552
PUFA, g	8.2 (4.9–12.3)	9.3 (5.4–12.8)	9.6 (5.9–12.0)	0.634
*N*-3 PUFA, g	0.1 (0.0–1.1)	0.2 (0.1–1.1)	0.3 (0.1–1.3)	0.150
*N*-6 PUFA, g	1.2 (0.4–5.3)	1.7 (0.7–3.3)	1.5 (0.7–4.2)	0.634
Carbohydrate, %	57.3 (50.7–62.4)	56.5 (48.7–61.3)	58.3 (50.9–64.4)	0.421
Fat, %	27.5 ± 8.6	28.2 ± 7.4	26.2 ± 8.3	0.373
Protein, %	14.6 (12.7–16.8)	15.2 (13.2–17.4)	14.6 (12.8–15.8)	0.198
*N*-3/*N*-6	0.1 (0.1–0.4)	0.1 (0.1–0.3)	0.2 (0.1–0.4)	0.440

MUFA, monounsaturated fatty acid; PUFA, polyunsaturated fatty acid; *N*-3, omega-3; *N*-6, omega-6; RAE, retinol activity equivalents. T1: K-KIDMED score Tertile 1 (range: −2 to 1), T2: K-KIDMED score Tertile 2 (range: 2 to 4), and T3: K-KIDMED score Tertile 3 (range: 5 to 8). Data are expressed as mean ± standard deviation (SD) or median (interquartile range). The *p*-value was calculated by one-way analysis of variance or the Kruskal–Wallis test. Statistically significant *p*-values are indicated in bold.

**Table 3:** Agreement between K-KIDMED and transfer of food intake data from the FFQ.

K-KIDMED	Scoring	FFQ	Kappa
Q1. I consume a fruit or fruit juice every day	‘yes’ response scores +1 point	1 point is given based on the consumption of a fruit or fruit juice every day	0.310 (0.193, 0.427)
Q2. I consume a fruit or fruit juice more than twice a day	‘yes’ response scores +1 point	1 point is given based on FFQ calculation if ≥2 portions of a fruit or fruit juice per day	0.144 (0.029, 0.259)
Q3. I eat fresh or cooked vegetables more than once a day (e.g., kimchi, cucumber, spinach salad, bean sprout salad, salad, etc.)	‘yes’ response scores +1 point	1 point is given based on FFQ calculation if ≥1 portion of vegetables per day	0.358 (0.222, 0.494)
Q4. I eat fresh or cooked vegetables more than twice a day	‘yes’ response scores +1 point	1 point is given based on FFQ calculation if ≥2 portions of vegetables per day	0.362 (0.243, 0.482)
Q5. I consume fish or seafood more than 2–3 times a week (e.g., mackerel, saury, salmon, flounder, flatfish, squid, shrimp, webfoot octopus, etc.)	‘yes’ response scores +1 point	1 point is given based on FFQ calculation if consumption of fish or seafood ≥ 2 times per week	0.148 (0.018, 0.277)
Q6. I eat fast food or instant food more than once a week(e.g., hamburgers, pizza, hot dogs, chicken, ramen, convenience store lunch boxes, etc.)	‘yes’ response scores −1 point.	−1 point is given based on FFQ calculation if consumption of fast food or instant food more than once per week	0.081 (−0.011, 0.173)
Q7. I eat foods containing beans more than once a week (e.g., tofu, soft tofu, bean sauce, soymilk, etc.)	‘yes’ response scores +1 point	1 point is given based on FFQ calculation if consumption of food containing beans more than once per week	0.352 (0.231, 0.473)
Q8. I eat multigrain rice, rye bread, barley bread, etc., more than 5 times a week	‘yes’ response scores +1 point	1 point is given based on FFQ calculation if consumption of food containing various grains ≥ 5 times per week	0.405 (0.280, 0.529)
Q9. I eat nuts more than 2–3 times a week (e.g., peanuts, walnuts, almonds, pistachios, macadamia, etc.)	‘yes’ response scores +1 point	1 point is given based on FFQ calculation if consumption of food containing nuts ≥ 2 times per week	0.186 (0.039, 0.332)
Q10. I eat more than two yogurts or two slices of cheese a day	‘yes’ response scores +1 point	1 point is given based on FFQ calculation if consumption of ≥1 portion of dairy products per day	0.072 (−0.018, 0.162)
Q11. I eat sweet snacks more than twice a day (e.g., beverages, snacks, candy, jelly, chocolate, etc.)	‘yes’ response scores −1 point.	−1 point is given based on FFQ calculation if consumption of ≥2 portions of sweet snacks per day	0.255 (0.141, 0.368)
Total score			

FFQ, Food Frequency Questionnaire; Kappa, Cohen’s kappa coefficient.

## Data Availability

Data are available under permission of corresponding author.

## Supplementary Materials

The following supporting information can be downloaded at: https://www.mdpi.com/article/10.3390/nu16162754/s1, Figure S1. The proportion of “yes” responses for each KIDMED item across different age groups; Figure S2. The proportion of “yes” responses for each KIDMED item according to presence of overweight.; Figure S3. The proportion of “yes” responses for each KIDMED item according to presence of obesity; Table S1. The K-KIDMED test used in this study; Table S2. The Food frequency questionnaire used in this study; Table S3. Nutritional characteristics of the study population according to the K-KIDMED question; Table S4. Nutritional characteristics of the study population according to the FFQ score tertile.
