# Supplementary material for: Development and Validation of a Questionnaire to Measure Adherence to a Mediterranean-Type Diet in Youth
Source: Nutrients. 2024 Aug 18;16(16):2754. doi: 10.3390/nu16162754 (PMC11356957; doi:10.3390/nu16162754)
Supplement: Supplementary file 1 [file nutrients-16-02754-s001.zip › nutrients-3137450-supplementary.pdf]

Supplementary Materials

Development and Validation of a Questionnaire to Measure Adherence to a Mediterranean-Type Diet in Youth

Yu-Jin Kwon [1], Young-Hwan Park [2], Yae-Ji Lee [3], Li-Rang Lim [4] and Ji-Won Lee [4,5,*]

[1] Department of Family Medicine, Yongin Severance Hospital, College of Medicine, Yonsei University, Yongin 16995, Republic of Korea; digda3@yuhs.ac
[2] Incheon Grand Internal Medicine Clinic, Incheon 22184, Republic of Korea; withimok@naver.com
[3] Department of Biostatistics and Computing, Yonsei University, Seoul 03722, Republic of Korea; ysbiostat@yuhs.ac
[4] Department of Family Medicine, Severance Hospital, College of Medicine, Yonsei University, Seoul 03722, Republic of Korea; erang0422@yuhs.ac
[5] Institute for Innovation in Digital Healthcare, Yonsei University, Seoul 03722, Republic of Korea
* Correspondence: indi5645@yuhs.ac

Food groups

Fruits	Strawberry, Tomato, Cherry Tomato, Oriental Melon, Watermelon, Peach<br>Grape, Apple, Pear, Persimmon, Dried Persimmon, Tangerine, Banana, Orange<br>Kiwi, Fruit Juice
Vegetables	Seaweed Soup, Kimchi Stew, Stir-fried Kimchi, Bean Sprouts (Seasoned, Soup), Mung Bean Sprouts, Seasoned Spinach, Bellflower Root (Raw, Seasoned), Pumpkin (Seasoned, Pancake), Seasoned Vegetables (excluding Bracken, Deodeok, and Eggplant), Cucumber (Raw, Fresh), Radish (Raw, Pickled, Dried), Vegetable Salad, Seasoned Green Onion, Seasoned Chives<br>Wrap Vegetables (Lettuce, Perilla Leaves, Cabbage, Pumpkin Leaves), Green Chili, Boiled Broccoli, Boiled Cabbage, Garlic (excluding Seasoned), Napa Cabbage Kimchi, Other Kimchi, Fresh Kimchi (excluding Napa Cabbage Kimchi), Pickled Vegetables (Chili, Garlic, Perilla Leaves, Onion, Radish), Cucumber Pickles, Lotus Root, Burdock Root, Stir-fried Mushrooms, Roasted Seaweed, Fresh Seaweed, Seasoned Seaweed, Seasoned Laver, Seasoned Seaweed Stems

Fish and Seafood	Loach Soup, Pollack Stew, Spicy Seafood Soup, Dried Pollack Soup, Mackerel, Pacific Saury (Grilled, Braised), Hairtail, Croaker (Grilled, Braised)<br>Anchovies, Stir-fried Anchovies, Squid (Raw, Boiled, Stir-fried), Dried Squid (Stir-fried, Seasoned), Salted Seafood, Salted Shrimp, Salted Squid, Salted Clams, Fish Cake (Stir-fried, Soup)
Fast Food and Instant Food	Ramen, Cup Ramen, Pizza, Hamburger, Sandwich, Ham, Fried Chicken
Soy and Tofu Products:	Soybean Paste Soup, Soybean Paste Stew, Fermented Soybean Stew, Tofu Stew, Soft Tofu Stew, Tofu, Braised Tofu, Pan-fried Tofu, Braised Soybeans, Soy Milk
Grains	Mixed Grain Rice (including Bean Rice)
Nuts	Peanuts, Chestnuts
Dairy Products	Milk (Regular, Low-fat), Liquid Yogurt , Spoonable Yogurt
Sweet Snacks:	Coffee, Carbonated Drinks (Cola, Cider, Fruit-flavored Soda), Malt Drink, Sweet Rice Drink, Snack Chips, Cookies, Crackers , Chocolate, Ice Cream, Frozen Desserts

Supplementary Table S1. The K-KIDMED test used in this study

	K-KIDMED	Yes	No
Q1	I consume a fruit or fruit juice every day		
Q2	I consume a fruit or fruit juice more than twice a day		
Q3	I eat fresh or cooked vegetables more than once a day. (Kimchi, cucumber, spinach salad, bean sprout salad, salad, etc.)		
Q4	I eat fresh or cooked vegetables more than twice a day		
Q5	I consume fish or seafood more than 2–3 times a week (for example, mackerel, saury, salmon, flounder, flatfish, squid, shrimp, webfoot octopus, etc.)		
Q6	I eat fast food or instant food more than once a week.<br>(for example, hamburgers, pizza, hot dogs, chicken, ramen, convenience store lunch boxes, etc.)		
Q7	I eat foods containing beans more than once a week. (for example, tofu, soft tofu, bean sauce, soymilk, etc.)		
Q8	I eat multigrain rice, rye bread, barley bread, etc. more than 5 times a week		
Q9	I eat nuts more than 2–3 times a week (for example, peanuts, walnuts, almonds, pistachios, macadamia, etc.)		
Q10	I eat more than two yogurts or two slices of cheese a day.		
Q11	I eat sweet snacks more than twice a day (for example, beverages, snacks, candy, jelly, chocolate, etc.)		

If the response to questions 1, 2, 3, 4, 5, 7, 8, 9, or 10 is 'yes,' 1 point is awarded. Conversely, a 'yes' response to questions 6 or 11 results in a deduction of 1 point.

Supplementary Table S2. The Food frequency questionnaire used in this study

Frequency of Consumption (times) / Food items	Rarely	1month		1week			1day			Standard Serving Size	Average Consumption Per Serving			
		1	2~3	1	2~4	5~6	1	2	3					
1. Steamed White Rice	①	②	③	④	⑤	⑥	⑦	⑧	⑨	1 bowl (300ml, B1T)	① ½	② 1	③ 1½	④ 2
2. Mixed Grain Rice (including Bean Rice)	①	②	③	④	⑤	⑥	⑦	⑧	⑨	1 bowl (300ml, B1T)	① ½	② 1	③ 1½	④ 2
3. Bibimbap, Fried Rice	①	②	③	④	⑤	⑥	⑦	⑧	⑨	1serving (외식제공량=500ml)	① ½	② 1	③ 1½	
4. Gimbap (Korean Sushi Roll)	①	②	③	④	⑤	⑥	⑦	⑧	⑨	1roll (=삼각김밥 2개)	① ½	② 1	③ 1½	④ 2
5. Curry Rice	①	②	③	④	⑤	⑥	⑦	⑧	⑨	1serving (외식제공량=500ml)	① ½	② 1	③ 1½	
6. Ramen, Cup Ramen	①	②	③	④	⑤	⑥	⑦	⑧	⑨	1piece	① ½	② 1	③ 1½	
7. Noodles (Kalguksu, Udon)	①	②	③	④	⑤	⑥	⑦	⑧	⑨	1serving (외식제공량=1000ml)	① ½	② 1	③ 1½	
8. Jajangmyeon, Jjamppong (Black Bean Noodles, Spicy Seafood Noodles)	①	②	③	④	⑤	⑥	⑦	⑧	⑨	1serving (외식제공량=1000ml)	① ½	② 1	③ 1½	
9. Cold Noodles (Naengmyeon)	①	②	③	④	⑤	⑥	⑦	⑧	⑨	1serving (외식제공량=1000ml)	① ½	② 1	③ 1½	
10. Rice Cake Soup (Tteokguk)	①	②	③	④	⑤	⑥	⑦	⑧	⑨	1serving (외식제공량=1000ml)	① ½	② 1	③ 1½	
11. Dumplings (Steamed or Fried)	①	②	③	④	⑤	⑥	⑦	⑧	⑨	1serving (외식제공량=만두6개)	① ½	② 1	③ 1½	
12. Sliced Bread	①	②	③	④	⑤	⑥	⑦	⑧	⑨	2slices	① 1	② 2	③ 3	
12-1. Butter, Margarine	①	②	③	④	⑤	⑥	⑦	⑧	⑨	2ts (10ml)	① 1	② 2	③ 3	
12-2. Jam	①	②	③	④	⑤	⑥	⑦	⑧	⑨	2ts (10ml)	① 1	② 2	③ 3	
13. Sweet Red Bean Bread, Steamed Buns (Hoppang), Cream Bread	①	②	③	④	⑤	⑥	⑦	⑧	⑨	1piece	① ½	② 1	③ 2	
14. Castella, Cake, Choco Pie	①	②	③	④	⑤	⑥	⑦	⑧	⑨	1 piece (조각)	① ½	② 1	③ 2	
15. Pizza	①	②	③	④	⑤	⑥	⑦	⑧	⑨	2 piece (½F3 × 2)	① 1	② 2	③ 3	
16. Hamburger, Sandwich	①	②	③	④	⑤	⑥	⑦	⑧	⑨	1serving (외식제공량)	① ½	② 1	③ 1½	
17. Baekseolgi, Sirutteok, Injeolmi, Jeolpyeon (Traditional Korean Rice Cakes)	①	②	③	④	⑤	⑥	⑦	⑧	⑨	1/2 piece	① ¼	② ½	③ 1	
18. Spicy Rice Cakes (Tteokbokki)	①	②	③	④	⑤	⑥	⑦	⑧	⑨	1cup (200ml)	① ½	② 1	③ 1½	
19. Cereal	①	②	③	④	⑤	⑥	⑦	⑧	⑨	1bowl (250ml, D1B, 우유포함)	① ½	② 1	③ 1½	

Frequency of Consumption (times)<br>Food items	Rar ely	1month		1we ek			1da y			Standard Serving Size	Average Consumption Per Serving		
		1	2~3	1	2~4	5~6	1	2	3				
20.Seolleongtang, Gomtang, Beef Bone Soup	①	②	③	④	⑤	⑥	⑦	⑧	⑨	1 bowl (250ml, D1B)	① ½	② 1	③ 1½
21.Gamjatang (Spicy Pork Bone Soup)	①	②	③	④	⑤	⑥	⑦	⑧	⑨	1 bowl (250ml, D1B)	① ½	② 1	③ 1½
22.Chueotang (Loach Soup)	①	②	③	④	⑤	⑥	⑦	⑧	⑨	1 bowl (250ml, D1B)	① ½	② 1	③ 1½
23.Dongtae Jjigae, Seafood Spicy Stew	①	②	③	④	⑤	⑥	⑦	⑧	⑨	1 bowl (250ml, D1B)	① ½	② 1	③ 1½
24.Seaweed Soup (Miyeokguk)	①	②	③	④	⑤	⑥	⑦	⑧	⑨	1 bowl (250ml, D1B)	① ½	② 1	③ 1½
25.Beef Soup, Yukgaejang, Radish Soup	①	②	③	④	⑤	⑥	⑦	⑧	⑨	1 bowl (250ml, D1B)	① ½	② 1	③ 1½
26.Dried Pollock Soup (Bugeoguk)	①	②	③	④	⑤	⑥	⑦	⑧	⑨	1 bowl (250ml, D1B)	① ½	② 1	③ 1½
27.Soybean Paste Soup (Doenjang Guk)	①	②	③	④	⑤	⑥	⑦	⑧	⑨	1 bowl (250ml, D1B)	① ½	② 1	③ 1½
28.Doenjang Jjigae, Cheonggukjang Jjigae (Soybean Paste Stews)	①	②	③	④	⑤	⑥	⑦	⑧	⑨	1cup (200ml)	① ½	② 1	③ 1½
29.Kimchi Stew, Stir-Fried Kimchi	①	②	③	④	⑤	⑥	⑦	⑧	⑨	1cup (200ml)	① ½	② 1	③ 1½
30.Budae Jjigae (Army Stew)	①	②	③	④	⑤	⑥	⑦	⑧	⑨	1cup (200ml)	① ½	② 1	③ 1½
31.Tofu Stew, Soft Tofu Stew (Sundubu Jjigae)	①	②	③	④	⑤	⑥	⑦	⑧	⑨	1cup (200ml)	① ½	② 1	③ 1½
32.Tofu, Braised Tofu, Pan-Fried Tofu	①	②	③	④	⑤	⑥	⑦	⑧	⑨	½cup (100ml)	① ¼	② ½	③ 1
33.Braised Soybeans (Kongjorim)	①	②	③	④	⑤	⑥	⑦	⑧	⑨	1TS (15ml)	① ½	② 1	③ 1½
34.Fried Eggs, Rolled Omelet	①	②	③	④	⑤	⑥	⑦	⑧	⑨	1piece (=달걀말이 4조각)	① ½	② 1	③ 2
35.Boiled Eggs, Steamed Eggs (Gyeranjjim)	①	②	③	④	⑤	⑥	⑦	⑧	⑨	1piece (=달걀찜 ½컵)	① ½	② 1	③ 2
36.Grilled Pork Belly (Samgyeopsal)	①	②	③	④	⑤	⑥	⑦	⑧	⑨	1serving (150g=1컵)	① ½	② 1	③ 2
37.Boiled Pork (Suyuk, Bossam)	①	②	③	④	⑤	⑥	⑦	⑧	⑨	1cup (200ml)	① ½	② 1	③ 1½
38.Stir-Fried Pork, Pork Bulgogi, Grilled Pork Ribs, Braised Pork Ribs	①	②	③	④	⑤	⑥	⑦	⑧	⑨	1cup (200ml)	① ½	② 1	③ 1½
39.Pork Sweet and Sour (Tangsuyuk), Pork Cutlet (Donkatsu)	①	②	③	④	⑤	⑥	⑦	⑧	⑨	1cup (200ml)	① ½	② 1	③ 1½
40.Grilled Beef	①	②	③	④	⑤	⑥	⑦	⑧	⑨	1serving (150g=1cup)	① ½	② 1	③ 2
41.Beef Bulgogi	①	②	③	④	⑤	⑥	⑦	⑧	⑨	1cup (200ml)	① ½	② 1	③ 1½
42.Ham	①	②	③	④	⑤	⑥	⑦	⑧	⑨	¼cup[(50ml)	① ⅛	② ¼	③ ½
43.Sundae (Korean Blood Sausage)	①	②	③	④	⑤	⑥	⑦	⑧	⑨	½cup (100ml)	① ¼	② ½	③ 1
44.Samgyetang (Ginseng Chicken Soup)	①	②	③	④	⑤	⑥	⑦	⑧	⑨	1serving(외식제공량=800ml)	① ½	② 1	③ 1½
45.Stir-Fried Chicken (Dakgalbi), Braised Chicken (Dakdoritang)	①	②	③	④	⑤	⑥	⑦	⑧	⑨	2cup (400ml)	① 1	② 2	③ 3
46.Fried Chicken	①	②	③	④	⑤	⑥	⑦	⑧	⑨	2piece (400ml=가식부200ml)	① 1	② 2	③ 3

47.Grilled Duck	①	②	③	④	⑤	⑥	⑦	⑧	⑨	1serving (150g=1컵)	① ½	② 1	③ 1½
48.Grilled or Braised Mackerel, Pacific Saury	①	②	③	④	⑤	⑥	⑦	⑧	⑨	¼cup (50ml, 가식부만)	① ⅛	② ¼	③ ½
49.Grilled or Braised Hairtail, Croaker	①	②	③	④	⑤	⑥	⑦	⑧	⑨	¼cup (50ml, 가식부만)	① ⅛	② ¼	③ ½
50.Anchovies, Stir-Fried Anchovies	①	②	③	④	⑤	⑥	⑦	⑧	⑨	**1TS** (15ml)	① **1ts**	② **1TS**	③ **¼C**
51.Squid (Raw, Boiled, Stir-Fried), Dried Squid (Stir-Fried, Seasoned)	①	②	③	④	⑤	⑥	⑦	⑧	⑨	¼ **cup** (=½컵, 100ml)	① ⅛	② ¼	③ ½
52.Marinated Crab (Ganjang Gejang)	①	②	③	④	⑤	⑥	⑦	⑧	⑨	**1TS** (15ml, 가식부만)	① **1ts**	② **1TS**	③ **¼C**
53.Fermented Shrimp, Fermented Squid, Fermented Clams	①	②	③	④	⑤	⑥	⑦	⑧	⑨	**1ts** (5ml)	① ½	② **1**	③ **3**
54.Fish Cake (Stir-Fried, Soup)	①	②	③	④	⑤	⑥	⑦	⑧	⑨	**1/2 cup** (100ml=국250ml,D1B)	① ¼	② ½	③ **1**

Frequency of Consumption (times) / Food items	Rarely	1month		1week			1day			Standard Serving Size	Average Consumption Per Serving		
		1	2~3	1	2~4	5~6	1	2	3				
55. Bean Sprouts (Seasoned or in Soup), Soybean Sprouts	①	②	③	④	⑤	⑥	⑦	⑧	⑨	**1/4 cup** (50ml=국250ml,D1B)	① ⅛	② ¼	③ ½
56.Spinach (Seasoned)	①	②	③	④	⑤	⑥	⑦	⑧	⑨	**¼ cup** (50ml)	① ⅛	② ¼	③ ½
57.Bellflower Root (Fresh or Seasoned)	①	②	③	④	⑤	⑥	⑦	⑧	⑨	**¼ cup** (50ml)	① ⅛	② ¼	③ ½
58. Zucchini (Seasoned or Pancake)	①	②	③	④	⑤	⑥	⑦	⑧	⑨	**¼ cup** (50ml)	① ⅛	② ¼	③ ½
59.Fernbrake, Crown Daisy, Eggplant, and Other Seasoned Vegetables	①	②	③	④	⑤	⑥	⑦	⑧	⑨	**¼ cup** (50ml)	① ⅛	② ¼	③ ½
60. Cucumber (Fresh or Pickled)	①	②	③	④	⑤	⑥	⑦	⑧	⑨	**¼ cup** (50ml)	① ⅛	② ¼	③ ½
61.Radish (Fresh or Pickled, Dried Radish)	①	②	③	④	⑤	⑥	⑦	⑧	⑨	**¼ cup** (50ml)	① ⅛	② ¼	③ ½
62.Vegetable Salad	①	②	③	④	⑤	⑥	⑦	⑧	⑨	**½ cup** (100ml)	① ¼	② ½	③ **1**
63. Green Onion Salad, Chive Salad	①	②	③	④	⑤	⑥	⑦	⑧	⑨	**¼ cup** (50ml)	① ⅛	② ¼	③ ½
64. Lettuce Wraps (Lettuce, Perilla Leaves, Cabbage, Pumpkin Leaves), Green Chili Peppers	①	②	③	④	⑤	⑥	⑦	⑧	⑨	10 Leaves of Lettuce	① **5**	② **10**	③ **15**
65. Boiled Broccoli, Boiled Cabbage	①	②	③	④	⑤	⑥	⑦	⑧	⑨	¼cup (50ml)	① ⅛	② ¼	③ ½
66.Garlic (Excludes Seasoned Variants)	①	②	③	④	⑤	⑥	⑦	⑧	⑨	2cloves (=⅓뿌리)	① **1**	② **2**	③ **3**
67. Ssamjang (Mixed Bean Paste and Chili Paste), Red Chili Paste	①	②	③	④	⑤	⑥	⑦	⑧	⑨	**2ts** (10ml)	① **1**	② **2**	③ **3**
68. Napa Cabbage Kimchi	①	②	③	④	⑤	⑥	⑦	⑧	⑨	¼cup (50ml)	① ⅛	② ¼	③ ½
69. Other Kimchi, Fresh Kimchi	①	②	③	④	⑤	⑥	⑦	⑧	⑨	¼cup (50ml)	① ⅛	② ¼	③ ½
70.Pickled Vegetables (Chili Peppers, Garlic, Perilla Leaves, Onions, Radish), Cucumber Pickles	①	②	③	④	⑤	⑥	⑦	⑧	⑨	**1TS** (15ml)	① ½	② **1**	③ **1½**
71.Braised Lotus Root, Braised Burdock Root	①	②	③	④	⑤	⑥	⑦	⑧	⑨	¼cup (50ml)	① ⅛	② ¼	③ ½
72. Pancakes (Chive Pancake, Kimchi Pancake, etc.)	①	②	③	④	⑤	⑥	⑦	⑧	⑨	½**slice** (C11 × ½)	① ¼	② ½	③ **1**
73.Japchae (Stir-Fried Noodles with Vegetables)	①	②	③	④	⑤	⑥	⑦	⑧	⑨	½cup (100ml)	① ¼	② ½	③ **1**
74.Stir-Fried Mushrooms	①	②	③	④	⑤	⑥	⑦	⑧	⑨	¼cup (50ml)	① ⅛	② ¼	③ ½
75.Roasted Seaweed, Fresh Seaweed, Seasoned Seaweed	①	②	③	④	⑤	⑥	⑦	⑧	⑨	1slice (=자른김 8장)	① ½	② **1**	③ **2**
76.Seasoned Sea Lettuce, Seaweed Salad	①	②	③	④	⑤	⑥	⑦	⑧	⑨	¼cup (50ml)	① ⅛	② ¼	③ ½
77.Stir-Fried Seaweed Stems	①	②	③	④	⑤	⑥	⑦	⑧	⑨	¼cup (50ml)	① ⅛	② ¼	③ ½
78.Stir-Fried Potatoes, Braised Potatoes	①	②	③	④	⑤	⑥	⑦	⑧	⑨	¼cup (50ml)	① ⅛	② ¼	③ ½

79.Steamed Potatoes, Roasted Potatoes	①	②	③	④	⑤	⑥	⑦	⑧	⑨	1	① ½	② 1	③ 2
80.Steamed Sweet Potatoes, Roasted Sweet Potatoes	①	②	③	④	⑤	⑥	⑦	⑧	⑨	1	① ½	② 1	③ 2
81.Steamed Corn, Roasted Corn	①	②	③	④	⑤	⑥	⑦	⑧	⑨	1	① ½	② 1	③ 1½
82.Milk (Regular, Low-Fat)	①	②	③	④	⑤	⑥	⑦	⑧	⑨	1cup (200ml)	① ½	② 1	③ 1½
Liquid Yogurt	①	②	③	④	⑤	⑥	⑦	⑧	⑨	1 (80ml)	① 소1 (65ml)	② 중1 (80ml)	③ 대1 (150ml)
Spoonable Yogurt	①	②	③	④	⑤	⑥	⑦	⑧	⑨	1 (100g)	① ½	② 1	③ 2
Soy Milk	①	②	③	④	⑤	⑥	⑦	⑧	⑨	1cup (200ml)	① ½	② 1	③ 1½

Frequency of Consumption (times) Food items		Rarely	1month		1week			1day			Standard Serving Size	Average Consumption Per Serving		
			1	2~3	1	2~4	5~6	1	2	3				
The following questions pertain to fruits. Please indicate whether you primarily consume the fruit during its peak season or regardless of the season, and then provide your average frequency of consumption. If you consume the fruit by blending it into a juice, it should be included in your fruit consumption frequency.														
○ Overall Fruit Consumption		①	②	③	④	⑤	⑥	⑦	⑧	⑨				
86. Strawberry	① Seasonal ② not	①	②	③	④	⑤	⑥	⑦	⑧	⑨	10 (=주스⅔컵)	① 5	② 10	③ 15
87. Tomato, Cherry Tomato	① Seasonal ② not	①	②	③	④	⑤	⑥	⑦	⑧	⑨	1(C7=방울토마토30개 =주스1컵)	① ½	② 1	③ 2
88. Cantaloupe	① Seasonal ② not	①	②	③	④	⑤	⑥	⑦	⑧	⑨	1 (O2)	① ⅓	② 1	③ 2
89. Watermelon	① Seasonal ② not	①	②	③	④	⑤	⑥	⑦	⑧	⑨	2 piece (F2 ×2)	① 1	② 2	③ 3
90. Peach	① Seasonal ② not	①	②	③	④	⑤	⑥	⑦	⑧	⑨	1 (C7)	① ½	② 1	③ 2
91. Grape	① Seasonal ② not	①	②	③	④	⑤	⑥	⑦	⑧	⑨	1cup (200ml, 포도알 컵에담아서)	① ½	② 1	③ 2
92. Apple	① Seasonal ② not	①	②	③	④	⑤	⑥	⑦	⑧	⑨	1 (C7=주스1컵)	① ½	② 1	③ 2
93. Pear	① Seasonal ② not	①	②	③	④	⑤	⑥	⑦	⑧	⑨	½ (C9 × ½)	① ¼	② ½	③ 1
94. Persimmon, Dried Persimmon	① Seasonal ② not	①	②	③	④	⑤	⑥	⑦	⑧	⑨	1 (C7)	① ½	② 1	③ 2
95. Tangerine	① Seasonal ② not	①	②	③	④	⑤	⑥	⑦	⑧	⑨	2 (C4 × 2)	① 1	② 2	③ 3
96. Banana	① Seasonal ② not	①	②	③	④	⑤	⑥	⑦	⑧	⑨	1 (=몽키바나나3개=주스1컵)	① ½	② 1	③ 2
97. Orange	① Seasonal ② not	①	②	③	④	⑤	⑥	⑦	⑧	⑨	1 (C7=주스1컵)	① ½	② 1	③ 2
98. Kiwi	① Seasonal ② not	①	②	③	④	⑤	⑥	⑦	⑧	⑨	2 (C4 × 2)	① 1	② 2	③ 3
99. Coffee		①	②	③	④	⑤	⑥	⑦	⑧	⑨	2ts (10ml, 믹스 1/1/1ts)	① 1	② 2	③ 3
99-1. If you consume coffee more than 3 times a day, how many times on average do you drink it per day? ____ Times per day														
99-2. Creamer		①	②	③	④	⑤	⑥	⑦	⑧	⑨	2ts (10ml)	① 1	② 2	③ 3

99-3. Sugar	①	②	③	④	⑤	⑥	⑦	⑧	⑨	2ts (10ml)	① **1**	② **2**	③ **3**
100. Green Tea	①	②	③	④	⑤	⑥	⑦	⑧	⑨	1cup (200ml)	① **½**	② **1**	③ **1½**
101. Carbonated Drinks (Cola, Lemon-lime Soda, Fruit-flavored Sodas)	①	②	③	④	⑤	⑥	⑦	⑧	⑨	1cup (200ml)	① **½**	② **1**	③ **1½**
102. Fruit Juice	①	②	③	④	⑤	⑥	⑦	⑧	⑨	1cup (200ml)	① **½**	② **1**	③ **1½**
103. Mixed Grain Drink, Sikhye (Sweet Rice Drink)	①	②	③	④	⑤	⑥	⑦	⑧	⑨	1cup (200ml)	① **½**	② **1**	③ **1½**
104. Snack Foods	①	②	③	④	⑤	⑥	⑦	⑧	⑨	1cup (200ml)	① **½**	② **1**	③ **1½**
105. Cookies, Crackers	①	②	③	④	⑤	⑥	⑦	⑧	⑨	6pieces	① **3**	② **6**	③ **9**
106. Chocolate	①	②	③	④	⑤	⑥	⑦	⑧	⑨	1/2	① **¼**	② **½**	③ **1**
107. Ice Cream, Frozen Desserts	①	②	③	④	⑤	⑥	⑦	⑧	⑨	1 (100ml)	① **½**	② **1**	③ **2**
108. Peanuts	①	②	③	④	⑤	⑥	⑦	⑧	⑨	¼cup (50ml)	① **⅛**	② **¼**	③ **½**
109. Chestnuts	①	②	③	④	⑤	⑥	⑦	⑧	⑨	3	① **1**	② **3**	③ **5**

Supplementary Table S3. Nutritional characteristics of the study population according to the K-KIDMED question

	Q1			Q2			Q3			Q4			Q5			Q6		
Characteristic	yes	no	p	yes	no	p	yes	no	p	yes	no	p	yes	no	p	yes	no	p
N	125	101		50	176		161	65		82	144		102	124		157	69	
Total energy, kcal	1,677.0 ± 510.5	1,733.3 ± 507.4	ns	1,749.5 ± 545.8	1,688.3 ± 498.4	ns	1,718.9 ± 474.8	1,659.2 ± 588.2	ns	1,693.9 ± 495.4	1,706.7 ± 518.1	ns	1,710.3 ± 465.6	1,695.1 ± 543.7	ns	1,751.0 ± 477.0	1,585.6 ± 564.3	*
Carbohydrate, g	230.6 ± 66.0	238.5 ± 72.5	ns	247.0 ± 62.1	230.4 ± 70.4	ns	236.8 ± 63.0	227.3 ± 82.1	ns	234.9 ± 70.8	233.6 ± 67.9	ns	235.6 ± 65.4	232.8 ± 71.8	ns	238.5 ± 67.2	223.7 ± 72.2	ns
Fat, g	51.4 (34.1-67.5)	52.4 (36.7-66.8)	ns	46.9 (32.6-68.1)	52.4 (36.7-67.2)	ns	51.0 (36.1-67.2)	53.0 (33.5-68.5)	ns	50.1 (35.9-67.4)	53.0 (35.7-66.5)	ns	51.3 (38.7-67.3)	52.9 (32.4-67.2)	ns	53.5 (38.7-67.5)	42.2 (32.5-63.1)	*
Protein (g)	61.9 (49.4-76.7)	62.1 (46.6-76.8)	ns	61.0 (44.9-79.0)	62.1 (48.7-76.3)	ns	61.0 (49.0-77.5)	63.1 (46.6-74.5)	ns	58.9 (48.7-72.3)	63.1 (48.2-81.7)	ns	61.9 (49.2-75.1)	62.1 (46.8-80.6)	ns	62.7 (50.9-80.8)	58.7 (43.8-71.0)	ns
Fiber (g)	15.8 (12.8-20.8)	14.4 (11.6-19.3)	ns	16.5 (13.6-22.7)	14.6 (11.9-19.3)	*	15.8 (12.7-20.7)	14.0 (10.9-17.4)	*	16.0 (12.7-20.8)	14.7 (12.1-19.3)	ns	16.0 (12.6-21.2)	14.6 (12.2-19.5)	ns	15.5 (12.5-20.0)	15.1 (12.1-19.8)	ns
Vit. A, ug RAE	243.5 (164.9-381.7)	306.4 (213.4-430.4)	ns	261.8 (166.7-429.9)	279.2 (182.0-415.6)	ns	278.1 (184.1-431.9)	268.7 (168.3-378.3)	ns	259.5 (167.9-376.6)	289.4 (179.1-441.2)	ns	260.6 (167.6-414.6)	280.7 (203.6-421.8)	ns	297.1 (194.7-423.1)	258.9 (153.9-362.0)	ns
Retinol, ug	89.2 (43.5-154.8)	118.7 (57.8-241.0)	*	81.5 (53.0-131.7)	113.0 (52.0-220.7)	ns	100.9 (51.1-167.0)	104.5 (53.3-238.7)	ns	81.5 (45.4-168.4)	107.8 (57.3-187.8)	ns	99.0 (56.8-158.9)	105.6 (51.5-212.5)	ns	115.7 (60.0-204.6)	75.1 (30.2-146.8)	*
β-Carotene, ug	1,625.4 (950.8-2,964.8)	1,640.8 (846.6-3,149.6)	ns	1,770.3 (951.7-3,791.2)	1,611.8 (839.6-2,886.4)	ns	1,761.9 (917.6-3,273.3)	1,292.4 (747.3-2,197.7)	*	1,490.6 (887.9-3,196.0)	1,702.8 (871.4-2,997.3)	ns	1,520.3 (908.1-2,886.4)	1,671.0 (828.2-3,356.3)	ns	1,521.6 (905.6-3,090.5)	1,838.0 (857.2-2,989.1)	ns
Vit. D, ug	2.5 (1.1-6.5)	2.6 (1.1-3.9)	ns	2.0 (0.9-7.5)	2.7 (1.1-4.6)	ns	2.2 (1.1-4.9)	3.1 (1.3-5.3)	ns	2.4 (0.9-5.1)	2.6 (1.1-4.9)	ns	2.5 (1.2-6.6)	2.6 (1.1-4.6)	ns	2.8 (1.1-5.2)	1.9 (0.5-3.4)	*
Vit. E, mg	12.2 (7.9-16.4)	12.3 (7.8-16.7)	ns	12.3 (8.8-16.1)	12.2 (7.8-16.8)	ns	12.4 (7.9-16.8)	12.1 (7.4-15.8)	ns	11.8 (7.8-16.6)	12.6 (8.2-16.4)	ns	12.2 (8.7-15.8)	12.4 (7.5-16.7)	ns	12.3 (8.4-16.4)	12.2 (7.2-16.6)	ns
Vit K. ug	69.8 (40.0-163.5)	58.7 (24.1-136.0)	ns	79.9 (44.3-224.7)	60.7 (33.6-127.6)	ns	69.1 (36.8-173.4)	57.1 (23.2-90.6)	ns	72.0 (38.5-150.5)	58.7 (27.8-152.0)	ns	70.2 (37.2-172.7)	58.4 (33.8-123.1)	ns	63.8 (35.2-152.0)	64.8 (33.9-144.7)	ns
Vit C, mg	39.3 (21.9-68.5)	32.4 (16.1-50.9)	*	43.0 (25.2-83.1)	33.9 (17.3-54.8)	*	37.1 (22.4-67.9)	28.1 (14.9-40.8)	*	37.7 (21.6-61.6)	33.9 (16.7-62.9)	ns	37.3 (22.4-68.3)	32.0 (16.4-56.4)	ns	34.2 (18.9-62.4)	36.3 (21.5-66.2)	ns
Thiamine, mg	1.3 (1.1-1.9)	1.4 (1.0-1.9)	ns	1.5 (1.1-1.9)	1.3 (1.0-1.9)	ns	1.4 (1.1-2.0)	1.3 (1.0-1.9)	ns	1.3 (1.1-1.8)	1.4 (1.0-2.0)	ns	1.4 (1.1-1.9)	1.3 (1.0-2.0)	ns	1.4 (1.1-1.9)	1.3 (0.9-1.9)	ns
Riboflavin, mg	1.3 ± 0.5	1.4 ± 0.6	ns	1.3 ± 0.5	1.3 ± 0.5	ns	1.3 ± 0.5	1.3 ± 0.6	ns	1.3 ± 0.5	1.3 ± 0.6	ns	1.3 ± 0.5	1.3 ± 0.6	ns	1.4 ± 0.5	1.2 ± 0.6	*
Niacin, mg	10.7 (8.5-13.9)	10.1 (7.3-13.1)	ns	9.7 (8.4-13.4)	10.9 (8.2-13.4)	ns	10.3 (8.3-13.1)	11.5 (8.0-14.6)	ns	9.8 (8.1-12.5)	10.9 (8.2-14.3)	ns	10.3 (8.4-12.8)	10.7 (7.5-14.5)	ns	10.6 (8.3-13.8)	10.3 (6.9-12.9)	ns
Vitamin B6, mg	1.2 (0.9-1.5)	1.2 (0.9-1.5)	ns	1.3 (1.0-1.8)	1.2 (0.9-1.5)	*	1.2 (0.9-1.7)	1.2 (0.8-1.4)	ns	1.2 (0.9-1.5)	1.3 (0.9-1.5)	ns	1.2 (1.0-1.7)	1.2 (0.8-1.5)	ns	1.2 (0.9-1.6)	1.2 (0.9-1.5)	ns
Folic acid, ug	319.9 (239.1-426.0)	320.5 (204.8-437.1)	ns	382.3 (274.6-449.8)	311.5 (208.8-426.1)	ns	334.8 (238.9-435.2)	284.4 (190.7-421.0)	ns	322.3 (237.5-431.6)	313.0 (206.6-426.8)	ns	330.1 (239.5-432.9)	309.1 (201.8-426.9)	ns	321.5 (237.4-427.0)	310.3 (184.8-430.8)	ns
Vit.B12, ug	4.3 (2.8-8.7)	3.9 (2.4-6.2)	ns	4.2 (2.7-9.5)	4.1 (2.6-6.6)	ns	4.1 (2.7-7.1)	4.2 (2.6-7.5)	ns	3.9 (2.8-6.7)	4.3 (2.6-7.8)	ns	3.9 (2.6-7.2)	4.3 (2.7-7.0)	ns	4.2 (2.7-7.4)	4.0 (2.3-6.6)	ns
Calcium, mg	388.9 (253.5-553.5)	415.6 (266.0-537.4)	ns	382.5 (241.3-510.0)	414.1 (265.5-541.9)	ns	392.2 (261.0-535.8)	414.1 (241.3-552.4)	ns	370.4 (254.0-496.2)	423.8 (262.5-561.2)	ns	427.4 (262.5-549.8)	395.6 (253.6-533.4)	ns	436.2 (283.7-562.3)	338.7 (225.0-462.5)	*
Phosphate, mg	956.5 ± 356.2	935.8 ± 334.9	ns	987.7 ± 381.7	935.7 ± 335.6	ns	967.0 ± 334.1	897.5 ± 373.6	ns	924.7 ± 328.0	960.5 ± 356.9	ns	956.9 ± 311.7	939.4 ± 373.5	ns	983.8 ± 345.5	860.5 ± 334.7	*
Sodium, mg	2,865.5 (2,298.4-3,969.5)	2,838.8 (2,213.0-3,993.1)	ns	2,805.2 (2,094.9-4,136.2)	2,936.8 (2,275.3-3,900.0)	ns	2,830.6 (2,283.0-4,082.2)	2,950.6 (2,036.6-3,688.5)	ns	2,776.2 (2,247.8-3,786.8)	2,985.7 (2,283.0-4,077.2)	ns	2,825.7 (2,332.5-3,967.6)	2,943.7 (2,206.3-4,005.4)	ns	3,028.1 (2,420.4-3,980.8)	2,419.0 (1,930.9-3,631.0)	*
Potassium, mg	2,102.8 (1,649.2-2,683.7)	1,978.7 (1,435.9-2,501.1)	ns	2,229.3 (1,719.9-2,686.4)	1,991.6 (1,493.5-2,513.3)	ns	2,030.9 (1,564.9-2,655.9)	1,981.1 (1,496.9-2,476.0)	ns	1,985.5 (1,561.4-2,600.0)	2,028.4 (1,567.9-2,513.3)	ns	1,990.2 (1,677.1-2,601.6)	2,051.8 (1,468.5-2,511.5)	ns	1,990.2 (1,500.8-2,702.5)	2,106.2 (1,675.1-2,477.0)	ns
Magnesium, mg	97.3 (58.7-134.6)	92.6 (57.7-127.3)	ns	112.0 (66.2-144.4)	93.0 (56.5-124.5)	ns	95.6 (59.5-134.6)	95.0 (52.7-120.1)	ns	99.3 (63.8-124.5)	92.6 (55.8-134.4)	ns	95.8 (58.8-139.7)	94.6 (57.5-120.5)	ns	95.6 (58.5-134.4)	94.7 (59.2-128.4)	ns
Iron, mg	11.2 (7.9-13.8)	12.0 (8.0-14.6)	ns	11.7 (8.0-13.7)	11.5 (7.9-14.2)	ns	11.6 (8.2-14.5)	11.1 (7.8-13.7)	ns	11.8 (7.8-13.5)	11.5 (8.2-15.1)	ns	11.6 (8.3-15.2)	11.5 (7.8-13.9)	ns	11.4 (7.9-13.8)	11.8 (8.7-15.3)	ns
Zinc, mg	8.4 (6.5-11.2)	8.2 (5.3-10.1)	ns	9.0 (7.0-12.3)	8.2 (5.9-10.1)	*	8.5 (6.3-11.1)	8.0 (5.5-10.1)	ns	7.9 (6.3-10.1)	8.5 (5.8-11.1)	ns	8.5 (6.3-11.2)	8.3 (6.0-10.1)	ns	8.5 (6.3-10.9)	7.7 (5.8-10.1)	ns
Copper, ug	402.6 (274.8-491.7)	416.3 (274.6-574.8)	ns	419.4 (275.5-547.1)	404.4 (276.1-524.6)	ns	421.0 (274.7-552.5)	364.6 (275.8-465.9)	ns	415.0 (270.5-561.7)	403.4 (280.8-514.1)	ns	416.8 (276.1-514.1)	378.7 (272.8-562.9)	ns	393.1 (270.4-524.6)	424.3 (277.8-544.4)	ns

Cholesterol, mg	241.9 (117.3-374.1)	267.7 (131.3-400.9)	ns	304.8 (109.9-395.6)	249.0 (124.3-373.3)	ns	248.7 (121.8-380.6)	267.2 (120.5-414.7)	ns	251.6 (108.4-375.5)	255.8 (128.6-391.9)	ns	242.5 (116.7-373.3)	260.9 (125.0-396.2)	ns	267.7 (147.1-391.7)	221.9 (91.6-373.1)	*
Saturated fatty acid, g	9.3 (5.7-14.0)	9.4 (5.7-14.5)	ns	9.1 (6.0-16.4)	9.5 (5.7-13.7)	ns	9.7 (5.7-14.9)	8.7 (5.8-13.6)	ns	9.8 (6.1-15.5)	8.7 (5.5-13.7)	ns	9.6 (5.7-14.9)	8.5 (5.7-14.1)	ns	9.5 (5.8-14.3)	9.0 (5.0-12.9)	ns
MUFA, g	9.3 (5.4-14.2)	8.9 (5.9-13.8)	ns	10.6 (6.0-18.0)	8.9 (5.6-13.9)	ns	9.4 (5.9-14.3)	8.6 (5.2-13.7)	ns	9.5 (6.1-15.6)	8.8 (5.3-13.7)	ns	9.1 (5.9-13.6)	9.3 (5.2-14.6)	ns	8.9 (5.9-13.9)	9.9 (5.2-14.1)	ns
PUFA, g	9.2 (4.9-12.7)	8.5 (5.1-12.9)	ns	8.8 (5.7-13.4)	8.7 (4.9-12.3)	ns	9.3 (5.3-13.4)	8.2 (3.9-11.0)	ns	9.6 (6.2-12.8)	8.5 (4.8-12.7)	ns	8.9 (4.8-12.8)	8.7 (5.9-12.7)	ns	8.7 (5.2-13.2)	9.4 (3.9-12.5)	ns
N-3 PUFA, g	0.3 (0.1-1.3)	0.2 (0.0-1.0)	ns	0.5 (0.1-1.9)	0.2 (0.1-1.1)	*	0.3 (0.1-1.1)	0.1 (0.0-1.3)	ns	0.4 (0.1-1.1)	0.2 (0.0-1.2)	ns	0.3 (0.1-1.3)	0.2 (0.1-1.1)	ns	0.2 (0.1-1.2)	0.2 (0.1-1.2)	ns
N-6 PUFA, g	1.5 (0.5-4.0)	1.8 (0.6-4.4)	ns	1.6 (0.8-3.9)	1.5 (0.5-4.2)	ns	1.7 (0.7-4.3)	1.0 (0.3-3.3)	*	1.7 (0.7-4.1)	1.3 (0.5-4.3)	ns	1.5 (0.5-3.9)	1.6 (0.5-4.3)	ns	1.5 (0.5-4.5)	1.6 (0.7-3.1)	ns

Abbreviations: MUFA, monounsaturated fatty acid; PUFA, polyunsaturated fatty acid; N-3, omega-3; N-6, omega-6

Data was expressed by mean ± standard deviations (SD) or median (interquartile range, IQR).

P-value was calculated by Student's t-test or Mann-Whitney U test.

Supplementary Table S3. Nutritional characteristics of the study population according to the K-KIDMED question-continued

	Q7			Q8			Q9			Q10			Q11		
**Characteristic**	yes	no	p	yes	no	p	yes	no	p	yes	no	p	yes	no	p
**N**	140	86		77	149		45	181		45	181		107	119	
Total energy, kcal	1,695.4 ± 469.6	1,713.0 ± 570.5	ns	1,730.2 ± 525.4	1,687.1 ± 501.0	ns	1,727.0 ± 501.9	1,695.7 ± 511.7	ns	1,692.4 ± 479.9	1,704.4 ± 516.9	ns	1,718.2 ± 485.8	1,687.3 ± 530.3	ns
Carbohydrate, g	232.8 ± 62.3	236.2 ± 78.9	ns	234.2 ± 73.8	234.1 ± 66.4	ns	238.9 ± 67.8	232.9 ± 69.3	ns	238.9 ± 64.0	232.9 ± 70.1	ns	241.4 ± 66.6	227.4 ± 70.5	ns
Fat, g	51.2 (36.7-65.8)	53.7 (32.4-72.3)	ns	51.5 (36.2-69.5)	51.8 (35.3-66.0)	ns	52.2 (36.2-68.7)	51.3 (35.3-66.9)	ns	50.6 (35.2-68.3)	52.2 (36.1-66.2)	ns	52.6 (36.7-66.9)	50.8 (34.3-68.9)	ns
Protein (g)	60.8 (50.1-72.7)	63.4 (46.3-82.3)	ns	62.2 (52.5-80.1)	61.6 (46.8-72.8)	ns	60.2 (51.5-75.3)	62.0 (46.8-77.1)	ns	54.4 (46.9-66.2)	63.0 (49.2-81.1)	*	58.1 (46.7-71.5)	66.1 (50.9-80.8)	ns
Fiber (g)	15.1 (12.5-19.9)	15.5 (11.9-20.0)	ns	16.1 (12.7-21.7)	14.8 (11.8-19.6)	ns	17.4 (12.8-22.9)	14.8 (12.1-19.4)	*	16.3 (12.7-20.7)	15.0 (12.3-19.9)	ns	15.5 (12.0-20.6)	15.5 (12.6-19.7)	ns
Vit. A, ug RAE	295.7 (175.0-432.2)	252.3 (179.0-403.7)	ns	311.3 (182.0-431.6)	263.3 (174.7-393.4)	ns	243.1 (156.6-377.7)	278.1 (196.7-422.8)	ns	229.8 (159.9-334.8)	297.1 (205.9-434.6)	*	266.5 (189.3-434.5)	299.2 (171.7-381.2)	ns
Retinol, ug	96.6 (50.0-204.8)	107.3 (59.4-167.8)	ns	88.4 (56.8-169.2)	105.6 (51.5-203.5)	ns	88.4 (56.8-156.6)	103.7 (52.1-203.2)	ns	113.7 (53.5-184.2)	97.1 (52.1-179.0)	ns	111.3 (56.9-172.8)	91.2 (49.5-199.0)	ns
β-Carotene, ug	1,691.3 (889.8-3,067.0)	1,462.1 (869.3-3,027.0)	ns	2,159.7 (1,279.0-3,332.0)	1,365.9 (777.3-2,784.9)	*	1,403.9 (839.6-2,939.1)	1,696.3 (887.3-3,069.3)	ns	1,007.4 (818.8-1,913.0)	1,789.3 (906.8-3,204.4)	*	1,639.9 (945.9-3,003.7)	1,492.8 (839.6-3,090.5)	ns
Vit. D, ug	2.6 (1.1-5.3)	2.4 (1.1-4.9)	ns	2.0 (0.9-4.4)	2.9 (1.1-5.3)	ns	2.0 (1.2-6.6)	2.6 (1.1-4.6)	ns	2.5 (1.0-4.9)	2.6 (1.1-5.0)	ns	2.3 (1.0-4.8)	2.8 (1.1-5.1)	ns
Vit. E, mg	12.6 (8.5-17.7)	12.2 (7.2-15.1)	ns	12.6 (8.7-17.3)	12.2 (7.7-16.0)	ns	13.3 (6.3-17.9)	12.2 (8.3-16.0)	ns	12.4 (7.0-16.1)	12.2 (8.4-16.7)	ns	12.8 (9.0-17.4)	11.6 (7.5-15.0)	ns
Vit K. ug	70.2 (36.1-169.8)	54.2 (26.1-104.5)	ns	82.4 (48.9-182.5)	52.5 (27.0-119.9)	*	70.2 (38.0-166.8)	62.6 (33.8-148.1)	ns	60.4 (32.4-119.4)	68.0 (34.7-164.4)	ns	68.7 (37.2-179.6)	62.9 (34.4-124.8)	ns
Vit C, mg	37.6 (20.3-72.6)	32.1 (18.2-45.2)	ns	37.3 (23.7-62.9)	33.9 (16.1-62.1)	ns	38.6 (21.5-67.3)	34.2 (17.3-62.6)	ns	32.0 (21.1-49.7)	34.6 (18.5-64.0)	ns	36.2 (20.2-68.9)	33.4 (18.3-59.1)	ns
Thiamine, mg	1.4 (1.1-1.9)	1.3 (1.0-2.0)	ns	1.3 (1.0-2.0)	1.4 (1.0-1.9)	ns	1.3 (1.1-1.8)	1.4 (1.0-2.0)	ns	1.3 (1.1-1.9)	1.4 (1.0-1.9)	ns	1.3 (1.0-1.9)	1.4 (1.1-2.0)	ns
Riboflavin, mg	1.3 ± 0.5	1.3 ± 0.6	ns	1.3 ± 0.5	1.3 ± 0.6	ns	1.3 ± 0.6	1.3 ± 0.5	ns	1.3 ± 0.5	1.3 ± 0.5	ns	1.3 ± 0.5	1.3 ± 0.6	ns
Niacin, mg	10.9 (8.3-13.3)	9.8 (7.4-13.7)	ns	10.2 (7.5-13.3)	10.7 (8.2-13.4)	ns	10.9 (8.6-13.3)	10.4 (7.8-13.4)	ns	9.8 (7.2-12.1)	10.8 (8.3-13.9)	ns	10.2 (7.9-13.3)	10.8 (8.3-13.5)	ns
Vitamin B6, mg	1.2 (0.9-1.6)	1.2 (0.8-1.5)	ns	1.3 (0.9-1.6)	1.2 (0.9-1.5)	ns	1.3 (1.0-1.6)	1.2 (0.9-1.5)	ns	1.2 (1.0-1.4)	1.2 (0.9-1.6)	ns	1.1 (0.9-1.5)	1.3 (0.9-1.6)	ns
Folic acid, ug	330.1 (236.2-445.9)	303.5 (207.4-425.8)	ns	368.6 (242.5-445.6)	306.8 (207.1-419.2)	ns	313.0 (241.3-480.9)	320.7 (209.1-422.4)	ns	295.1 (214.4-426.0)	321.5 (231.6-437.1)	ns	312.3 (227.9-448.9)	325.5 (223.5-418.5)	ns
Vit.B12, ug	4.1 (2.8-7.0)	4.1 (2.4-7.4)	ns	3.8 (2.6-5.6)	4.5 (2.7-8.7)	*	3.9 (3.1-8.9)	4.2 (2.6-7.0)	ns	3.9 (2.8-7.5)	4.2 (2.6-7.0)	ns	3.8 (2.6-7.2)	4.5 (2.7-7.0)	ns
Calcium, mg	410.3 (255.9-545.8)	392.3 (252.8-531.7)	ns	414.1 (292.3-541.9)	395.6 (246.0-538.0)	ns	384.3 (292.3-538.6)	405.9 (253.6-546.6)	ns	406.7 (312.3-539.2)	396.7 (249.5-540.3)	ns	400.2 (257.4-560.4)	402.5 (253.3-535.7)	ns
Phosphate, mg	944.7 ± 322.6	951.7 ± 384.2	ns	967.3 ± 356.6	936.8 ± 341.4	ns	983.0 ± 359.0	938.3 ± 343.4	ns	899.1 ± 327.7	959.2 ± 350.5	ns	929.0 ± 349.9	964.0 ± 343.6	ns
Sodium, mg	2,823.0 (2,256.9-3,830.4)	3,280.0 (2,277.0-4,101.8)	ns	2,881.1 (2,283.0-4,139.7)	2,842.5 (2,206.3-3,816.4)	ns	2,950.6 (2,472.4-4,165.4)	2,829.2 (2,092.9-3,816.4)	ns	2,849.6 (2,351.3-3,699.2)	2,849.5 (2,213.0-4,047.9)	ns	2,814.4 (2,214.1-3,545.9)	2,936.8 (2,282.9-4,165.4)	ns
Potassium, mg	1,990.2 (1,578.4-2,582.6)	2,024.4 (1,472.9-2,557.5)	ns	2,291.4 (1,716.3-2,708.2)	1,971.4 (1,461.0-2,478.3)	*	2,068.9 (1,677.1-2,618.0)	2,003.0 (1,550.9-2,542.8)	ns	1,925.8 (1,499.9-2,449.5)	2,025.3 (1,570.4-2,579.4)	ns	1,989.5 (1,438.9-2,462.3)	2,068.9 (1,576.7-2,687.7)	ns
Magnesium, mg	95.8 (58.7-134.7)	92.5 (58.4-124.6)	ns	114.3 (80.5-155.2)	81.4 (52.0-116.2)	*	112.5 (62.8-144.8)	92.8 (57.4-124.8)	ns	96.4 (52.2-125.2)	95.0 (60.1-134.7)	ns	97.3 (58.5-134.1)	92.6 (59.0-131.4)	ns
Iron, mg	11.9 (8.4-14.8)	10.9 (7.6-13.0)	ns	12.8 (10.3-17.6)	10.8 (7.3-13.2)	*	12.3 (8.9-14.1)	11.0 (7.8-14.2)	ns	10.6 (7.8-13.0)	11.6 (8.2-14.9)	ns	11.6 (7.6-13.7)	11.5 (8.3-14.9)	ns
Zinc, mg	8.4 (6.4-10.8)	8.0 (5.6-11.0)	ns	8.5 (7.1-11.4)	8.1 (5.7-10.5)	*	8.7 (6.2-10.3)	8.3 (6.0-10.9)	ns	7.4 (6.1-9.7)	8.5 (6.1-11.2)	ns	8.1 (6.0-10.1)	8.6 (6.3-11.3)	ns
Copper, ug	421.7 (301.8-523.7)	354.0 (257.5-533.7)	ns	434.4 (325.3-591.4)	372.6 (255.9-480.1)	*	425.2 (308.5-591.2)	397.5 (270.5-512.5)	ns	359.9 (250.4-465.9)	416.4 (289.3-560.0)	ns	402.6 (281.3-519.0)	413.4 (270.4-530.1)	ns
Cholesterol, mg	239.7 (114.3-388.7)	299.8 (131.3-386.4)	ns	202.2 (110.8-376.5)	267.4 (137.6-390.8)	ns	255.2 (108.0-419.0)	255.2 (127.5-373.1)	ns	224.9 (96.9-358.7)	266.0 (130.6-395.5)	ns	240.5 (123.3-383.6)	266.0 (114.3-390.9)	ns
Saturated fatty acid, g	9.8 (5.8-14.1)	8.3 (5.3-13.9)	ns	8.9 (5.8-13.7)	9.7 (5.5-14.2)	ns	9.7 (7.1-14.1)	9.4 (5.5-14.0)	ns	10.0 (7.4-15.6)	9.0 (5.3-13.7)	ns	9.9 (5.7-15.3)	8.9 (5.7-13.1)	ns
MUFA, g	9.4 (5.9-14.2)	8.6 (5.5-13.4)	ns	9.1 (5.1-12.8)	9.4 (6.0-14.8)	ns	10.4 (6.5-14.3)	8.8 (5.5-13.9)	ns	9.5 (6.1-14.9)	9.1 (5.5-13.7)	ns	9.3 (5.7-15.6)	9.2 (5.6-13.2)	ns
PUFA, g	9.5 (5.9-13.7)	7.7 (4.5-11.6)	*	9.3 (4.8-11.8)	8.7 (5.0-13.4)	ns	9.8 (4.7-14.7)	8.6 (5.2-12.2)	ns	9.0 (4.3-13.4)	8.7 (5.1-12.7)	ns	8.7 (4.9-13.4)	9.2 (5.4-12.3)	ns
N-3 PUFA, g	0.3 (0.1-1.4)	0.1 (0.0-0.9)	*	0.2 (0.1-1.0)	0.2 (0.1-1.3)	ns	0.5 (0.1-1.5)	0.2 (0.1-1.0)	ns	0.2 (0.1-1.1)	0.2 (0.1-1.2)	ns	0.2 (0.0-1.2)	0.2 (0.1-1.2)	ns

N-6 PUFA, g	1.8 (0.7-4.3)	1.0 (0.4-3.8)	*	1.7 (0.8-3.8)	1.4 (0.5-4.5)	ns	2.2 (0.6-5.8)	1.5 (0.5-3.9)	ns	1.3 (0.4-5.5)	1.6 (0.6-3.8)	ns	1.5 (0.5-3.8)	1.5 (0.6-4.6)	ns

Abbreviations: MUFA, monounsaturated fatty acid; PUFA, polyunsaturated fatty acid; N-3, omega-3; N-6, omega-6

Data was expressed by mean ± standard deviations (SD) or median (interquartile range, IQR).

P-value was calculated by Student’s t-test or Mann-Whitney U test.

Supplementary Table S4. Nutritional characteristics of the study population according to the FFQ score tertile.

Characteristic	T1, [-1,3]	T2, [4,4]	T3, [5,8]	p-value
n	118	37	71	
Total energy, kcal	1,656.6 ± 516.5	1,760.9 ± 499.0	1,746.3 ± 500.8	0.380
Carbohydrate, g	230.3 ± 74.8	234.0 ± 53.3	240.3 ± 66.2	0.627
Fat, g	51.3 (32.4-64.5)	54.6 (40.1-78.6)	51.5 (38.3-67.8)	0.443
Protein, g	60.2 (45.6-80.9)	65.9 (50.0-76.7)	63.0 (52.4-75.2)	0.384
Fiber, g	14.1 (10.9-17.6)	16.2 (12.9-21.9)	16.9 (13.3-21.8)	**0.002**
Vitamin A, μg RAE	258.5 (169.4-403.7)	299.9 (223.5-429.5)	315.3 (181.7-432.2)	0.277
Retinol, μg	100.9 (49.1-173.0)	113.5 (50.0-174.8)	97.1 (63.8-218.8)	0.558
β-Carotene, μg	1,520.9 (763.8-2,964.8)	1,697.1 (1,151.8-2,964.7)	1,644.8 (950.2-3,278.3)	0.323
Vitamin D, μg	2.2 (1.1-4.2)	2.0 (0.9-4.4)	3.3 (1.6-7.2)	**0.040**
Vitamin E, mg	11.9 (6.8-15.4)	11.8 (9.2-15.4)	14.1 (8.5-18.1)	0.115
Vitamin K, μg	50.1 (25.1-129.0)	75.5 (54.7-174.3)	75.3 (41.4-159.5)	0.066
Vitamin C, mg	28.8 (14.5-49.4)	41.7 (27.2-73.9)	39.9 (22.2-79.1)	**0.002**
Thiamine, mg	1.3 (1.0-1.9)	1.5 (1.1-2.1)	1.5 (1.1-1.9)	**0.039**
Riboflavin, mg	1.2 ± 0.5	1.3 ± 0.5	1.4 ± 0.5	0.052
Niacin, mg	10.4 (7.2-14.3)	11.1 (8.6-13.2)	10.3 (8.6-13.3)	0.780
Vitamin B6, mg	1.1 (0.8-1.5)	1.3 (1.0-1.7)	1.3 (1.0-1.6)	0.154
Folic acid, μg	284.3 (187.7-406.8)	365.1 (280.2-464.4)	382.8 (244.7-444.5)	**0.012**
Vitamin B12, μg	3.8 (2.4-6.3)	4.1 (2.9-8.7)	5.0 (3.0-8.9)	**0.040**
Calcium, mg	368.9 (238.1-493.4)	411.2 (301.0-565.4)	447.9 (314.5-605.1)	**0.025**
Phosphate, mg	904.9 ± 339.5	967.3 ± 376.5	1,006.6 ± 336.5	0.139
Sodium, mg	2,922.8 ± 1,266.4	3,382.1 ± 1,378.4	3,198.5 ± 1,283.7	0.119
Potassium, mg	1,845.0 (1,346.8-2,483.2)	2,260.6 (1,814.0-2,643.2)	2,301.2 (1,715.3-2,717.5)	**0.014**
Magnesium, mg	80.7 (55.3-114.5)	101.9 (56.6-140.6)	114.5 (74.6-140.1)	**0.007**
Iron, mg	10.5 (7.4-14.3)	11.7 (7.9-14.3)	12.3 (10.3-13.9)	0.145
Zinc, mg	7.5 (5.6-9.9)	8.8 (6.9-11.2)	9.1 (7.0-11.4)	**0.039**
Copper, ug	355.2 (249.0-478.8)	433.5 (332.6-593.6)	438.5 (337.8-585.9)	**0.009**
Total cholesterol, mg	248.9 (122.8-355.4)	242.4 (119.6-356.7)	299.5 (125.1-434.3)	0.375
Saturated fatty acid, g	8.3 (4.9-14.7)	9.7 (6.2-11.7)	9.9 (7.2-14.7)	0.292
MUFA, g	8.2 (5.1-13.6)	9.6 (5.9-13.3)	10.0 (6.6-15.1)	0.054
PUFA, g	8.1 (4.5-11.6)	9.8 (4.8-15.0)	9.9 (6.0-13.1)	0.095
N-3 PUFA, g	0.1 (0.0-1.0)	0.4 (0.1-1.2)	0.4 (0.1-1.6)	**0.016**
N-6 PUFA, g	1.2 (0.5-3.4)	1.8 (0.6-5.5)	1.9 (1.1-4.2)	0.164
Carbohydrate, %	57.4 (50.2-64.5)	56.0 (46.2-61.9)	56.1 (50.9-60.3)	0.486
Fat, %	27.0 ± 8.3	28.6 ± 8.8	28.0 ± 7.2	0.503
Protein, %	14.3 (12.3-17.1)	15.2 (13.5-16.7)	14.7 (13.4-16.1)	0.452
N-3/N-6	0.1 (0.1-0.3)	0.1 (0.1-0.4)	0.2 (0.1-0.5)	0.088

Supplementary Figure S1. The proportion of "yes" responses for each KIDMED item across different age groups

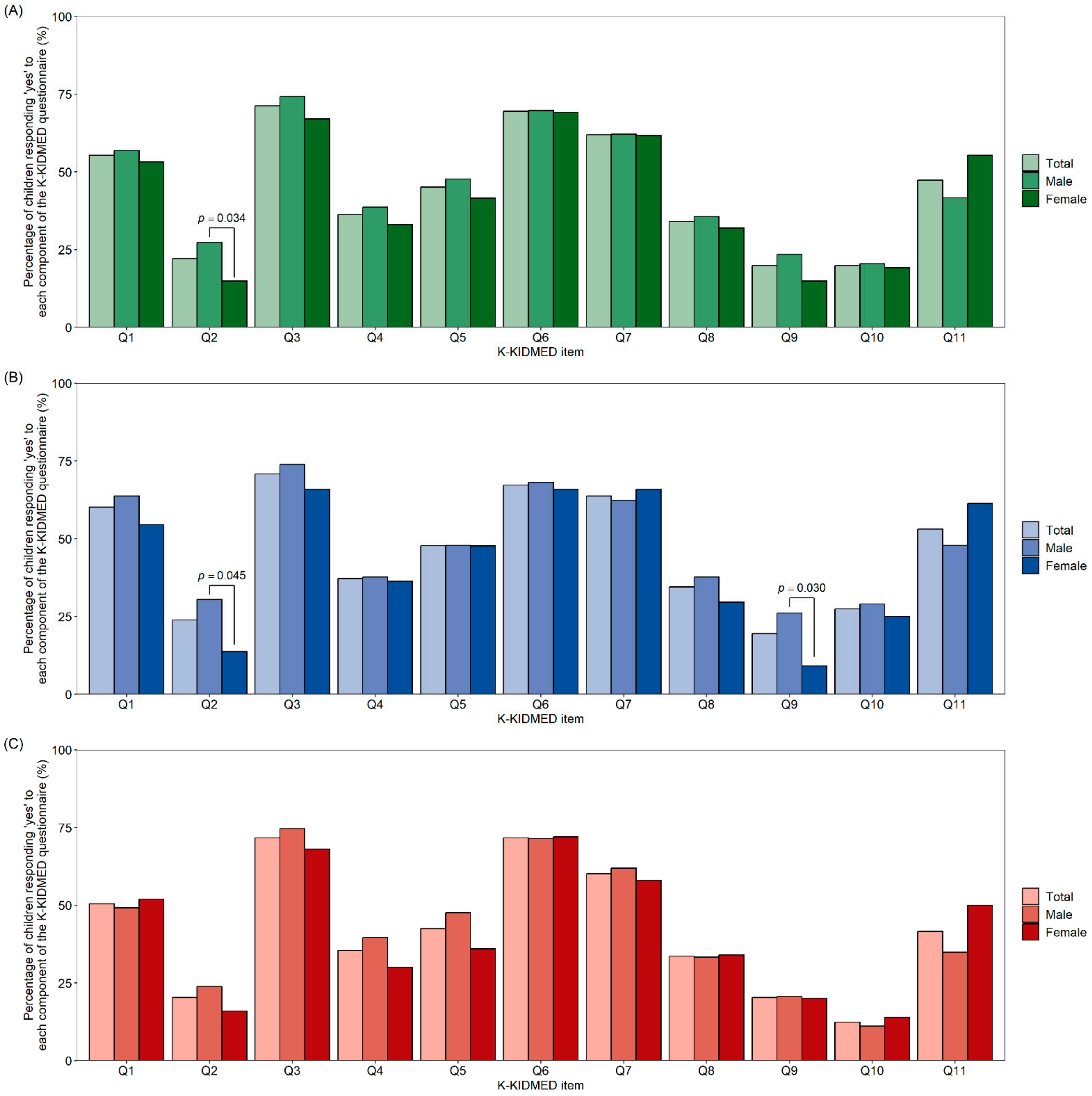

(A) The proportion of "yes" responses for each KIDMED item in the total sample per sex

(B) The proportion of "yes" responses for each KIDMED item per sex in the 5-9 years age group

(C) The proportion of "yes" responses for each KIDMED item per sex in the 10-12 years age group

Supplementary Figure S2. The proportion of "yes" responses for each KIDMED item according to presence of overweight.

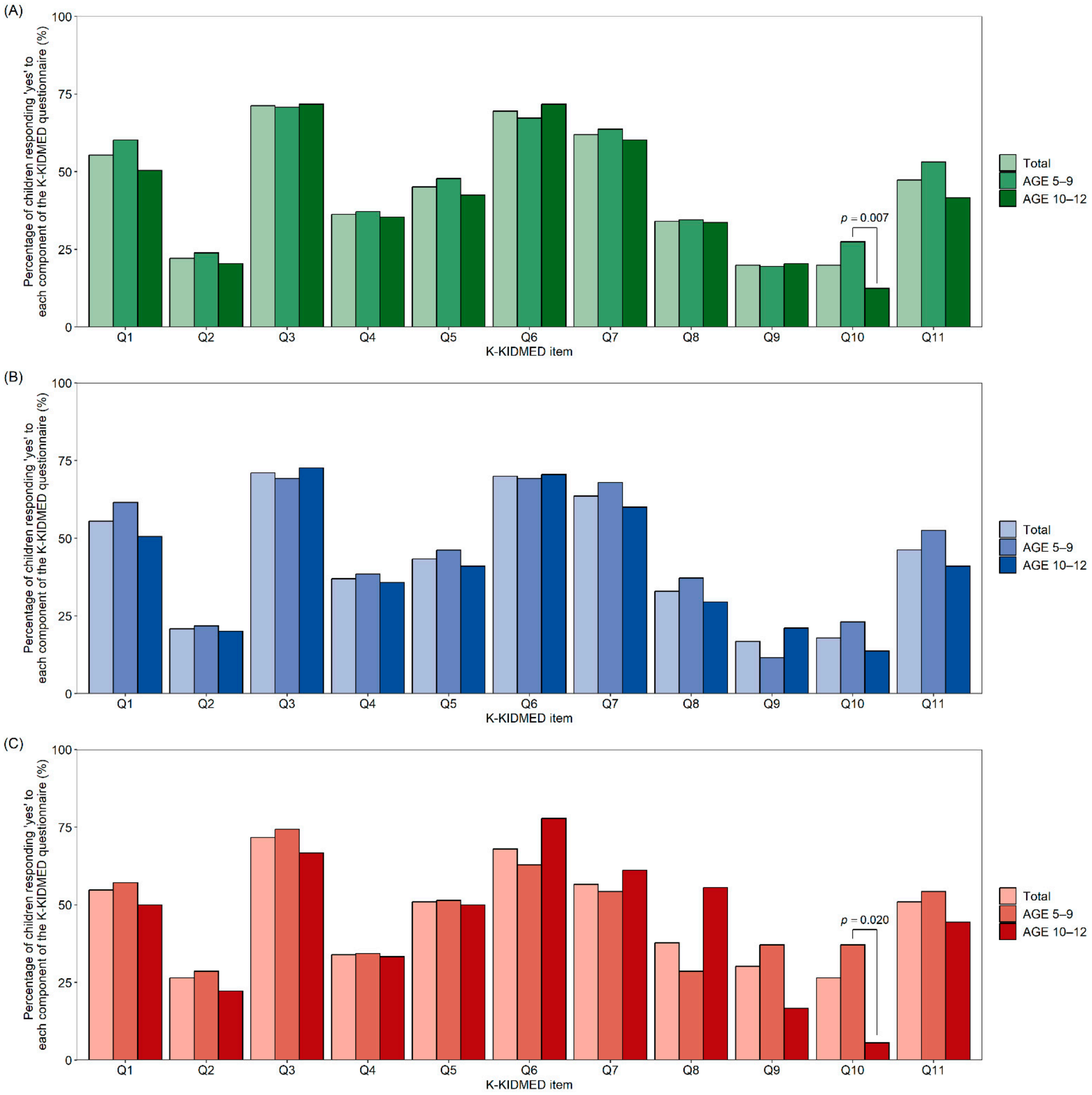

(A) The proportion of "yes" responses for each KIDMED item in the total sample and per age group

(B). The proportion of "yes" responses for each KIDMED item in non-overweight children per total sample and age group

(C). The proportion of "yes" responses for each KIDMED item in overweight children per total sample and age group

Supplementary Figure S3. The proportion of "yes" responses for each KIDMED item according to presence of obesity

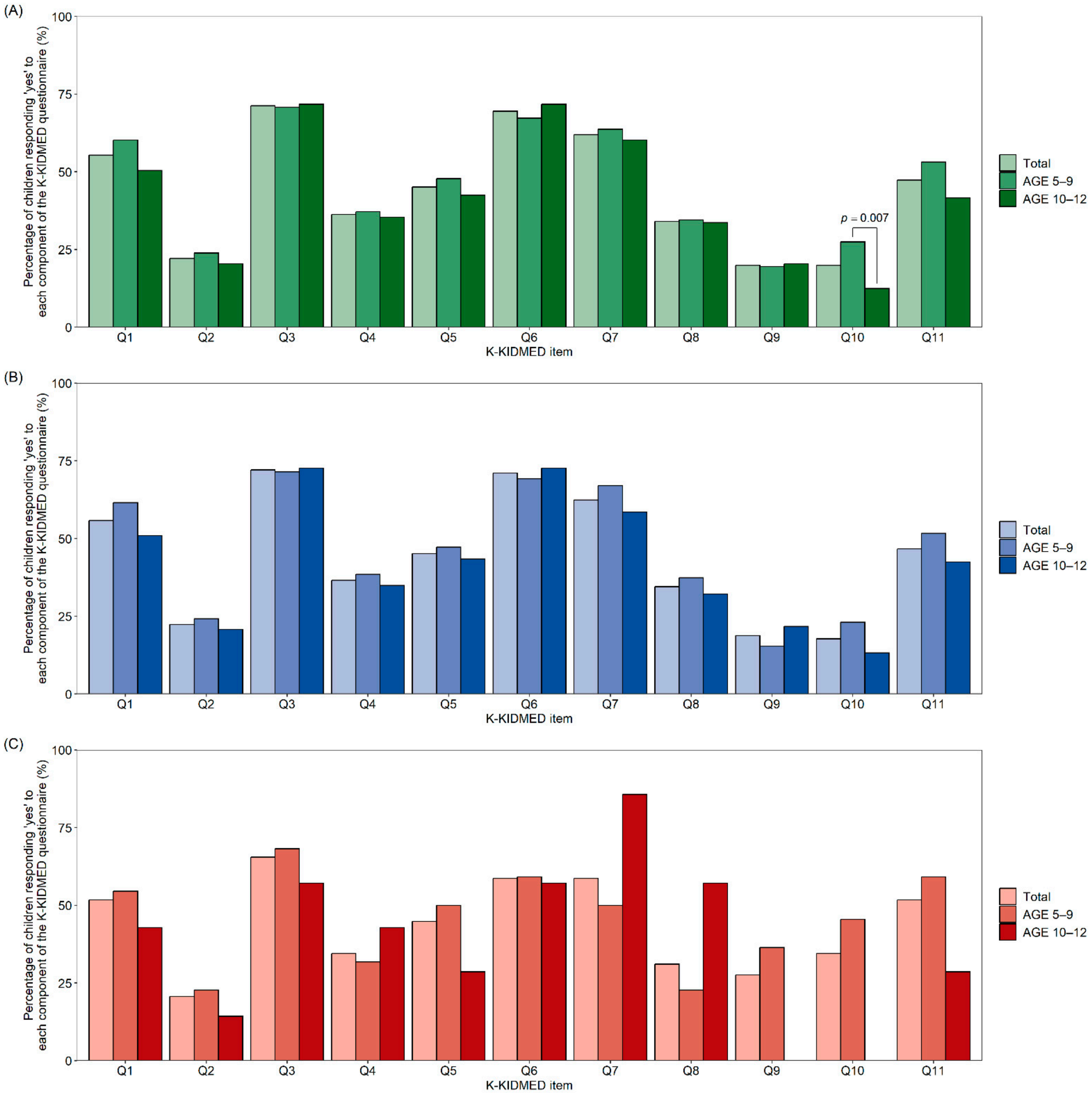

(A) The proportion of "yes" responses for each KIDMED item in the total sample and per age group

(B). The proportion of "yes" responses for each KIDMED item in non-obese children per total sample and age group

(C). The proportion of "yes" responses for each KIDMED item in obese children per total sample and age group
